# Pelagic calcium carbonate production and shallow dissolution in the North Pacific Ocean

**DOI:** 10.1038/s41467-023-36177-w

**Published:** 2023-02-20

**Authors:** Patrizia Ziveri, William Robert Gray, Griselda Anglada-Ortiz, Clara Manno, Michael Grelaud, Alessandro Incarbona, James William Buchanan Rae, Adam V. Subhas, Sven Pallacks, Angelicque White, Jess F. Adkins, William Berelson

**Affiliations:** 1 Universitat Autònoma de Barcelona, Institute of Environmental Science and Technology, Barcelona, Spain; 2 Catalan Institution for Research and Advanced Studies (ICREA), Barcelona, Spain; 3 Universitat Autònoma de Barcelona, BABVE Department, Barcelona, Spain; 4 Laboratoire des Sciences du Climat et de l’Environnement (LSCE/IPSL), Université Paris-Saclay, Gif-sur-Yvette, France; 5 University of St Andrews, School of Earth and Environmental Sciences, St Andrews, United Kingdom; 6 Centre for Arctic Gas Hydrate, Environment and Climate (CAGE), Department of Geosciences, UiT The Arctic University of Norway, Tromsø, Norway; 7 British Antarctic Survey, Natural Environmental Research Council, Cambridge, United Kingdom; 8 Università di Palermo, Dipartimento di Scienze della Terra e del Mare, Palermo, Italy; 9 Department of Marine Chemistry and Geochemistry, Woods Hole Oceanographic Institution, Woods Hole, MA USA; 10 School of Ocean and Earth Science and Technology, Department of Oceanography, University of Hawai’i at Manoa, Honolulu, USA; 11 Department of Geology and Planetary Sciences, Linde Center for Global Environmental Science, California Institute of Technology, Pasadena, CA USA; 12 University of Southern California, Department of Earth Sciences, Los Angeles, CA USA

**Keywords:** Carbon cycle, Marine chemistry

## Abstract

Planktonic calcifying organisms play a key role in regulating ocean carbonate chemistry and atmospheric CO_2_. Surprisingly, references to the absolute and relative contribution of these organisms to calcium carbonate production are lacking. Here we report quantification of pelagic calcium carbonate production in the North Pacific, providing new insights on the contribution of the three main planktonic calcifying groups. Our results show that coccolithophores dominate the living calcium carbonate (CaCO_3_) standing stock, with coccolithophore calcite comprising ~90% of total CaCO_3_ production, and pteropods and foraminifera playing a secondary role. We show that pelagic CaCO_3_ production is higher than the sinking flux of CaCO_3_ at 150 and 200 m at ocean stations ALOHA and PAPA, implying that a large portion of pelagic calcium carbonate is remineralised within the photic zone; this extensive shallow dissolution explains the apparent discrepancy between previous estimates of CaCO_3_ production derived from satellite observations/biogeochemical modeling versus estimates from shallow sediment traps. We suggest future changes in the CaCO_3_ cycle and its impact on atmospheric CO_2_ will largely depend on how the poorly-understood processes that determine whether CaCO_3_ is remineralised in the photic zone or exported to depth respond to anthropogenic warming and acidification.

## Abstract (web summary)

Ziveri et al find calcifying phytoplankton dominate pelagic CaCO_3_ production, but a large portion of this CaCO_3_ dissolves in the photic zone - they suggest the processes driving shallow CaCO_3_ dissolution are key to understanding the role of planktonic calcifiers in regulating atmospheric CO_2_.

## Introduction

The marine calcium carbonate (CaCO_3_) cycle is a key component of the global carbon cycle, and is intimately related to atmospheric CO_2_ (ref. ^1^). The formation of CaCO_3_ in the ocean is a process largely controlled by the biological calcification of marine organisms^2^. Planktonic calcifying organisms at the base of the food web (from primary producers to zooplankton) have played a key role since the Mesozoic^3^, via processes including regulation of surface water alkalinity, ballasting of organic matter and alkalinity export, and establishment of a pelagic carbonate buffer capable of influencing major CO_2_ change^4–6^.

Since the seminal work of Milliman^7–9^ on the production and accumulation of CaCO_3_ in the ocean, several studies have aimed to quantify total CaCO_3_ pelagic production^10–12^ and the contribution of specific calcifying plankton groups. However, the relative contribution of the main calcifying taxa to total CaCO_3_ pelagic production has not yet been directly quantified.

There is large uncertainty in total pelagic CaCO_3_ production, with current estimates varying between 0.7–4.7 Pg C yr^−1^ (0.6−3.9×10^14^ mol CaCO_3_ yr^−1^)^7,10–13^. In general, estimates based on satellite observations or modeling of ecosystems/carbonate chemistry in the surface ocean suggest higher CaCO_3_ production^10,12,13^ whereas estimates based on export from the production layer typically report lower values^7,8,11^.

There is also uncertainty on the make-up of pelagic CaCO_3_ production by different groups. Mainly based on sediment trap export fluxes and sediment data, there is a general understanding that coccolithophores (single-celled haptophytes inhabiting the photic zone, performing photosynthesis and producing calcite) and planktonic foraminifera (single-celled marine eukaryotes producing calcite) each contribute ~50% to the global pelagic CaCO_3_ production and sedimentation^8,14–16^. However, more recent papers have highlighted the potential of shelled pteropods (specialized free-swimming pelagic sea snails producing aragonite) as an important component of pelagic CaCO_3_ production^13,17,18^. Other taxa such as heteropods (holoplanktonic gastropods with aragonite shells, Pterotracheoidea) may also contribute to a lesser degree.

These planktonic calcifying taxa have specific mechanisms of biogenic calcification, and associated differences in vulnerability to ocean acidification^19,20^ with their shell solubility depending on their specific polymorph mineralogy and Mg content^21–23^. In addition, planktonic calcifiers exhibit a large range of particulate inorganic carbon to particulate organic carbon ratios (PIC/POC)^18,24,25^, which influences the integrated carbon export rain ratio, an important term for carbon cycling in the oceans and atmospheric CO_2_^1,26^. Furthermore, and as we discuss later, the association of PIC and POC together within calcifying organisms may play a critical role in driving CaCO_3_ dissolution above the saturation horizon^27^. However, despite the importance of pelagic calcification to the marine carbon cycle, key questions remain about pelagic CaCO_3_ production rates, standing stocks, and export fluxes. Most importantly, it is critical to determine the contributions of different planktonic calcifying groups to pelagic calcification, the proportion of aragonite versus calcite, and the magnitude of CaCO_3_ production compared to export.

The North Pacific Ocean is a key region for understanding the role of pelagic calcifiers in the global CaCO_3_ budget, due to its large volume and the wide range of biogeochemical conditions from the subtropical to subpolar gyres. In addition, the waters of the North Pacific are some of the most undersaturated in the global ocean with respect to calcite and aragonite, and thus calcifying organisms in the region are most at risk of future ocean acidification driven by anthropogenic CO_2_ emissions^28,29^. Although there are studies of the relative distribution of pelagic calcifiers in the North Pacific^30,31^, estimates of their relative contribution to CaCO_3_ standing stock and production rates are severely lacking. The discovery of excess alkalinity above the saturation horizon in the North Pacific has sparked debate about the role of different pelagic calcifiers and their contribution to the alkalinity budget above the thermodynamic saturation horizon^23,32,33^.

**Fig. 1:**
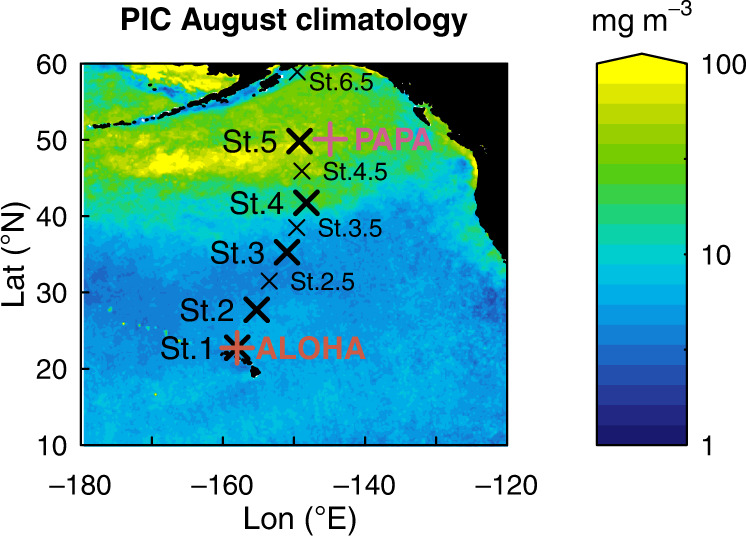
Satellite PIC and location map. August satellite-derived Particular Inorganic Carbon^97^ (PIC; mg CaCO_3_ m^-3^) climatology (2002-2017) and location of C-DisK-IV stations (black crosses) and long-term sediment trap studies (orange/pink crosses). Large black crosses show the location of Niskin bottle rosette, plankton tow, and floating sediment trap sampling sites at C-DisK-IV stations. Small black crosses show sites with additional plankton tow sampling only at C-DisK-IV stations. Note the logarithmic scale.

We conducted a research cruise from subtropical to subpolar North Pacific waters in which we assessed the pelagic living CaCO_3_ standing stock. We targeted the main planktonic calcifiers at five survey stations, from Honolulu, Hawaii, to Seward, Alaska, (Fig. 1, Tables S1 and S2). We deployed plankton nets to sample calcifying zooplankton and rosettes of Niskin bottles to target calcifying phytoplankton. In addition, four intermediate planktonic towing stations were sampled and integrated into the overall data set (Fig. 1, Tables S1, S2). Coccolithophores, foraminifera, pteropods, and heteropods were quantified and the CaCO_3_ biomass was estimated, providing the first overall picture of the total CaCO_3_ living standing stock (i.e. inventory), and the relative contribution of the main calcite and aragonite planktonic producers in the productive upper ocean. Using estimates of turnover time for each group we estimate annual production (Methods), and compare this to aragonite and calcite biomineral export out of the surface ocean to 100 and 200 m water depth estimated using floating sediment traps deployed during the time of sampling^34^ and historical time series in the region.

## Results and discussion

### North Pacific CaCO_3_ standing stocks

**Fig. 2:**
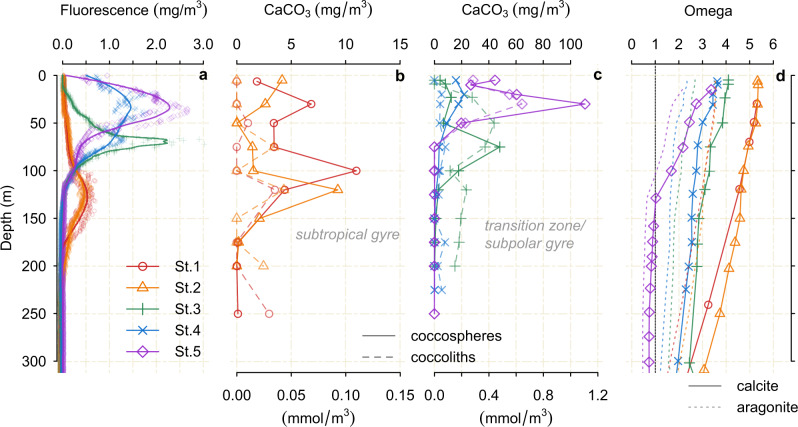
Coccolithophore standing stock vertical profiles. **a** chlorophyll fluorescence. Coccosphere, and coccolith CaCO_3_ from C-DisK-IV stations (**b**) 1 and 2 and, (**c**) 3, 4, and 5. Note the different x axis range on panels (**b**) and (**c**). **d** Omega calcite and aragonite at the five stations.

**Fig. 3:**
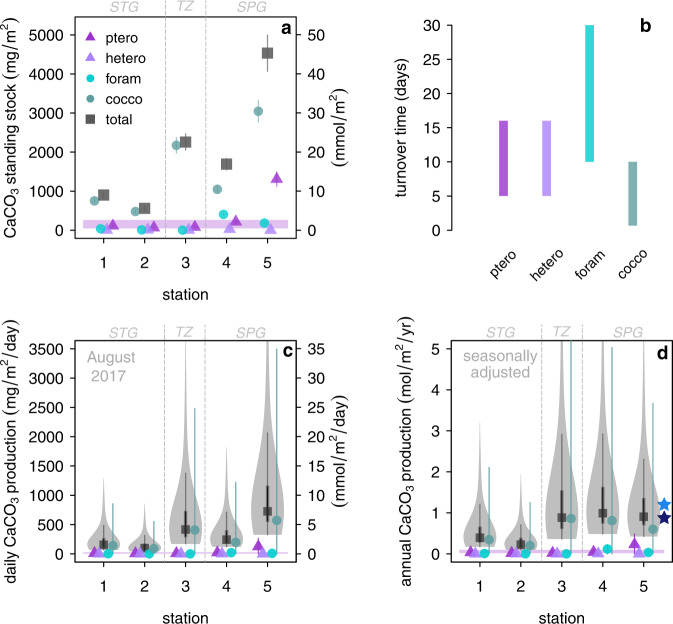
Standing stock and production by pelagic calcifiers. **a** living CaCO_3_ standing stock **b** turnover time of calcifying taxa used to calculate production from standing stock (the range represented by the bar length is applied with a flat probability distribution in our error propagation) **c** CaCO_3_ production per day (August 2017) **d** CaCO_3_ annual production corrected for seasonal bias using satellite-derived PIC/chlorophyll^97,104^ and zooplankton seasonality estimates (all data and metadata are publicly available at hahana.soest.hawaii.edu/hot/hot-dogs/interface.html). The total CaCO_3_ production is shown by the violin plots in panels (**c**) and (**d**), where the probability density of the estimate is represented by the thickness of the shaded area and the grey lines show the 68% and 95% confidence interval (CI); note the non-normal distribution with the high-tail on the upper estimate. Error bars for the standing stock (**a**) and production (**c**, **d**) by individual taxon represent the 95% CI (Methods). STG, TZ, and SPG represent subtropical gyre, transition zone, and subpolar gyre, respectively. Purple bands on panels **a**, **c**, and **d** show 68% range of pteropod standing stock and daily/annual production calculated using the MAREDAT database (Methods). The blue stars on panel **d** show the estimates of total production calculated with in-situ pH and fCO_2_ measurements at Ocean Station PAPA (ref. ^57^, light blue), and estimates of production in the subpolar North Pacific calculated using the seasonal cycle of alkalinity and dissolved inorganic carbon (ref. ^12^, dark blue). STG, TZ, and SPG represent subtropical gyre, transition zone, and subpolar gyre, respectively.

The total CaCO_3_ standing stock is lower in the nutrient-poor and less productive subtropical gyre (~560–900 mg m^-2^; note here and throughout we refer to mg of CaCO_3_, unless specified otherwise), and strongly increases into the nutrient-rich and productive subpolar gyre (~1700–4500 mg m^−^^2^ total) (Figs. 2 and 3a, Table S3), reflecting the major ecological shift across the North Pacific, from low-CaCO_3_ production in the oligotrophic subtropics, to high-CaCO_3_ production in the subpolar region^32,35^. We find that coccolithophores dominate the CaCO_3_ producing standing stock at all stations, demonstrating a mean contribution of ~79% (with a range of 62–96% across all sites) to the total CaCO_3_ standing stocks. Pteropods contribute ~14% (3–29% range), followed by foraminifera (~6% mean, 0.1–22% range), and heteropods (~1%, 0–2%). Calcite from coccolithophores and foraminifera is thus the most abundant mineral, constituting ~86% of the standing stock (71–96%), with aragonite making up ~14% of the standing stock (4–30%).

The coccosphere CaCO_3_ standing stock depth profiles follow the overall chlorophyll fluorescence, albeit with the scaling between fluorescence and coccosphere CaCO_3_ varying between stations, (Fig. 2, Fig. S1), indicating a substantial contribution by haptophytes/coccolithophores to the total standing stock of photosynthetic algae production^36^. We observe a shallowing of the chlorophyll maximum depth and coccolithophore CaCO_3_ standing stock maximum from subtropical to subpolar stations (Fig. 2).

Our estimates of living coccolithophore CaCO_3_ standing stocks range from 0.13 mg m^-3^ at 175 m (St. 1) in the subtropical gyre to 110 mg m^-3^ at 30 m (St. 5) in the subpolar gyre, with depth-integrated estimates from 753 mg m^-2^ to 3048 mg m^-2^ at the same stations, respectively (Fig. 2b, c, Tables S2 and S3). Our results support previous work in the North Pacific based on coccolithophore cell concentration, which showed that their biomass is highest at high latitudes, decreasing in temperate and subtropical regions^30^ (Fig. S8). The subpolar and transitional North Pacific Ocean is also known as a region of sustained seasonal *E. huxleyi* (the most abundant and cosmopolitan coccolithophore species) blooms^37^ with an estimated maximum satellite-derived PIC concentration of ~0.8 mmol m^−3^ (CaCO_3_ of 80 mg m^-3^) in August/September (note, satellite PIC is limited to retrievals over the first optical depth of satellite data, ~10 m). There is a remarkable agreement between our estimated values of coccolithophore CaCO_3_ from the shallowest sampled water depth (~5 m, i.e. the surface CaCO_3_ concentration) and satellite-derived PIC concentrations (Figs. S2 and S3), which are mostly tuned to capture coccolithophore PIC. This supports the high correlation between satellite-derived PIC and measured PIC surface water concentrations suggested by Balch and others;^10,38^ however as previously noted^38^ and discussed later, much of the coccolith PIC production can occur below the depth of the optical retrieval, particularly in the subtropics where the production layer deepens.

We also quantify the CaCO_3_ contribution of loose coccoliths (calcite plates extruded to the cell surface forming the coccosphere; Fig. 2b; Table S5). Coccoliths are shed into the surrounding waters following death and breakup of the coccosphere, or produced continuously by some species^38–40^. We found that loose coccolith CaCO_3_ can contribute significantly to the total CaCO_3_ standing stock in the productive photic layer, with maximum values of 44 and 64 mg m^-3^ in *E. huxleyi* blooms at Stations 3 and 5 (Fig. S3). Our results show loose coccolith distribution is tied to the distribution of intact coccospheres and PIC, as observed in previous studies^10,38^. We note that our coccolithophore living standing stock estimates (used to calculate CaCO_3_ production rate) only include whole coccosphere cells, and excludes loose coccoliths.

Our pteropod standing stock concentrations in the subpolar gyre range from 109–802 ind. m^-3^, broadly within the published range of the pteropod standing stocks in the northwestern Pacific (e.g.; ^41,42^, Fig. 3; Table S3; Fig. S5) and the Gulf of Alaska^43,44^, although pteropods show a significant seasonal and inter-annual variability in the coastal habitats of the Gulf of Alaska^43,44^. The recent study of Bednaršek et al.^45^ found that the abundance of pteropods collected in May 2015 in the subpolar gyre and Gulf of Alaska was (42–423 ind m^-2^), around two orders of magnitude lower than our abundances observed in August 2017 in the same region (Stations 4–6, Table S3) and previous studies^43^. This difference in abundance may relate to seasonal and inter-annual variability in this region, and/or the use of larger mesh size in their study (200–335 µm)^45^, which could have resulted in an underrepresentation of the small size pteropod (juvenile) fraction. Our estimates from the subtropical gyre (22–391 ind. m^-3^) (Table S3) are similar to previous estimates for this region and are higher than values observed across the Atlantic Ocean^46^.

Our pteropod CaCO_3_ concentrations range from 0.2–8.6 mg m^-3^ (Fig. 3a). We find good agreement between our estimates of pteropod CaCO_3_ standing and North Pacific sites in the MAREDAT database^17^ (Methods; Fig. 3), which show a typical concentration of 0.5 (0.2–1.1, 68% CI) mg m^-^^3^. Despite our pteropod CaCO_3_ standing stock results being similar to/higher than previous estimates from the North Pacific/North Atlantic, our values are lower than the proposed 23.17 mg CaCO_3_ m^-3^ global-mean value of shelled pteropod CaCO_3_ standing stock concentration^18^. However, our analysis shows that this global dataset is heavily skewed (Skewness = 13.3, where a value above one indicates a skewed dataset), with the median pteropod biomass value reported^18^ being around three orders of magnitude smaller than the reported mean. As such the global-mean value is not a useful descriptor of this data compilation^18^. Our analysis shows typical pteropod CaCO_3_ biomass globally is 0.3 (0.08–0.9, 68% CI) mg m^-3^ in the upper 250 m and 0.2 (0.07–0.8, 68% CI) mg m^-3^ in the upper 1000 m (Methods; Supplemental Fig. S6), two orders of magnitude lower than the global-mean value reported by ref. ^18^, and in line with our results from the North Pacific.

**Fig. 4:**
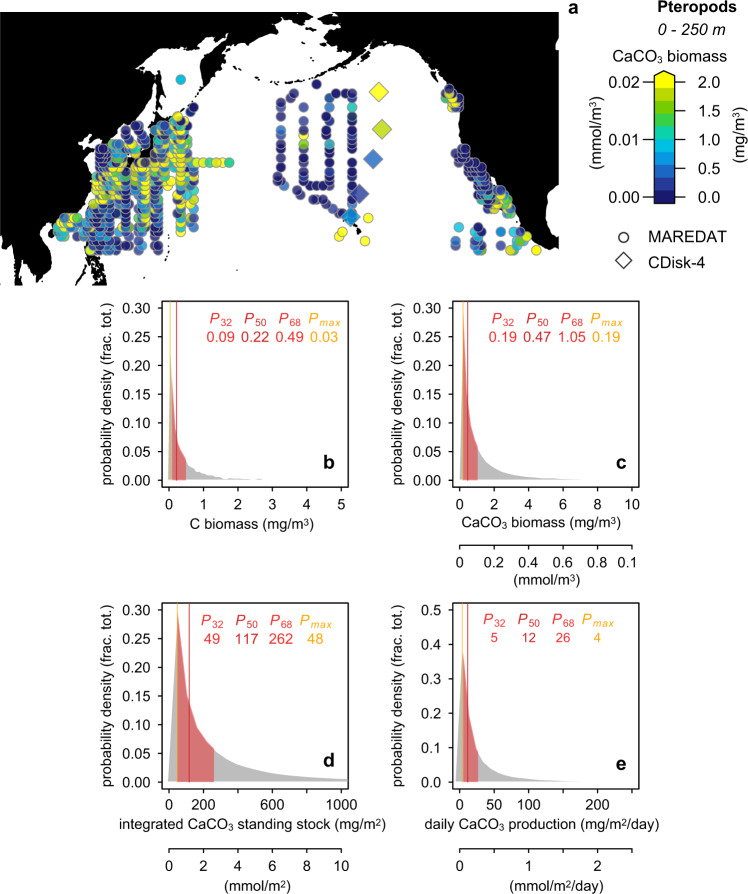
Pteropod CaCO_3_ biomass estimates. **a** Pteropod CaCO_3_ biomass estimated from the MAREDAT database^17^ and measured in this study; note, maximum values extend above 2 mg m^-^^3^. Probability density of pteropod (**b**) Carbon biomass (**c**) CaCO_3_ biomass (**d**) Integrated CaCO_3_ standing stock (**e**) daily CaCO_3_ production calculated using samples in the upper 250 m of the North Pacific from the comprehensive MAREDAT database^17,18^ (Methods). Red shading indicates 32–68% confidence interval range. Red values show the 32nd, 50th, 68th percentiles; orange value shows the value with the highest probability (all values given in mg). Note the distributions are highly skewed.

Our vertically integrated pteropod CaCO_3_ standing stocks range between ~64–111 mg m^-2^ in subtropical gyre and between ~215–1306 mg CaCO_3_ m^-2^ in the subpolar gyre (Fig. 3; Table S3; Fig. S5). We find good agreement between our estimates and the vertically integrated pteropod CaCO_3_ standing stock calculated from North Pacific sites in the MAREDAT database^17^ (Methods; Fig. 4), which show a typical value of 121 (50–270, 68%) mg m^-2^, with our estimates thus being slightly higher.

Our heteropod standing stock concentrations range from 5–40 ind. m^-3^ and 0.01–0.1 mg CaCO_3_ m^-3^, and their presence is limited to the subtropics and transition zone. Although previous estimates of heteropod standing stocks are extremely scarce, comparison to previous abundances from a latitudinal Atlantic Ocean transect confirmed that heteropods almost exclusively inhabit warm waters and the recorded maximum of 0.7 ind. m^-3^ (ref. ^46^) is lower than our estimates in the North Pacific. Our vertically integrated heteropod CaCO_3_ standing stocks range from 3-35 mg CaCO_3_ m^-2^; heteropods thus contribute between 3–12% of the total aragonite standing stock in the subtropics and transition zone, but are absent from the subpolar region.

Our estimates of integrated foraminiferal CaCO_3_ standing stock range from 9–37 mg m^-2^ in the subtropical gyre to 182–404 mg m^-2^ in the subpolar gyre. Although previous estimates of foraminiferal standing stock in the North Pacific are scarce, our estimates of the integrated vertical standing stock of the number of foraminifera from the subtropical gyre/transition zone sites are similar to, or slightly higher, than previous estimates of the integrated vertical standing stock from the subtropical gyre/transition zone in the western North Pacific^31,47^ (Figs. S4, 5). Our estimates of the vertically integrated standing stock of the number of foraminifera in the subpolar North Pacific (190,000–250,000 ind. m^-2^) are generally higher than the estimates of Taylor et al.^31^, which ranged up to ~80,000 ind. m^-2^, although such high values are not unprecedented, with previous estimates of the vertically integrated standing stock from the North Atlantic ranging up to ~390,000 ind. m^-2^ ref. ^48^.

### Pelagic CaCO_3_ production

We calculate CaCO_3_ production rate by dividing our measurements of the living CaCO_3_ standing stock by estimates of the turnover time (i.e. typical life span) of each group (Methods, Fig. 3b). Our approach assumes all of the organisms within the standing stock are living; this is valid for foraminifera, pteropods, and heteropods as individuals sink relatively quickly after death^47,49^, and the individuals sampled contained cytoplasm/soft tissue (Methods). For coccolithophores this assumption is valid as we only consider intact coccospheres, which disaggregate quickly upon death into the component coccoliths^50^, and is supported by the fact that the peaks of coccolithophore CaCO_3_ match the peaks in chlorophyll fluorescence (Fig. 2). We include the caveat that our approach assumes the living standing stock is in approximate steady state.

Given that coccolithophores have a shorter turnover time (1.5–10 days) than the other calcifying groups (Methods, Table 1, Fig. 3b) and dominate the CaCO_3_ standing stock, they account for ~86% (67–97%) of total CaCO_3_ annual production across the sites. Pteropods contribute ~10% to total production (2–17%), heteropods ~0.3% (0–1%), and foraminifera contribute ~2% (0.02–9%). As such, 89% of the CaCO_3_ production is calcite (70–97%), with the remainder being aragonite (Fig. 3c).

Given the large seasonality of PIC production^51,52^ (Fig. S7), we estimate annual CaCO_3_ production correcting for seasonal bias (Methods, Table S6). Our seasonally corrected annual CaCO_3_ production estimates range from 0.2–0.4 mol m^-2^ yr^-1^ in the subtropical gyre, a similar range or slightly lower than the estimate of the production rate of 0.7 mol m^-2^ yr^-1^ in the subtropical/tropical Atlantic^53^, although in good agreement with the global mean estimate of 0.4 mol m^-2^ yr^-1^ (ref. ^10^, Fig. 3d). Our estimates from the transition zone and the productive subpolar gyre are higher (0.9–1.0 mol m^-2^ yr^-1^) than this global average even at the 95% confidence interval (Fig. 3d); however, they agree well with the estimate of production calculated with in-situ pH and fCO_2_ measurements at Ocean Station PAPA through (1.2 mol m^−2^ yr^-1^; ref. ^54^), and estimates of production in the subpolar North Pacific calculated using the seasonal cycle of alkalinity and dissolved inorganic carbon (0.9 ± 0.1 mol m^-2^ yr^-1^; ref. ^12^).

To explore the implications of our estimates of CaCO_3_ production for global production, we use a global climatology of satellite-derived PIC (Fig. S7). While there is a high correlation between satellite PIC and our estimates of surface PIC concentration (Fig. S2a; ref. ^38^), our results indicate depth integrated CaCO_3_ production is only twice as high in the nutrient-rich subpolar gyre than the nutrient-poor subtropical gyre, smaller than the range expected from the satellite PIC (CaCO_3_) estimates, which suggest PIC concentrations ~6–7 times higher in the subpolar region compared to the subtropics. This difference likely reflects the deeper (coccolithophore) CaCO_3_ production in the subtropics, as well as the thickness of the coccolithophore productive layer (Fig. 2) from the upper ~175 m in the subtropics to the upper ~75 m in subpolar region, which will bias the satellite-derived PIC estimates to lower values^38^. For Stations 1–4 surface PIC is below 10 mg m^- ^^3^ (both at the time of sampling and in the annual mean climatology), yet we observe a depth integrated seasonally corrected production of 0.2–1 mol m^-^^2^ yr^-1^ at these sites (Table S6). Note, this surface PIC value is very similar to the threshold proposed by Balch et al.^38^ (0.13 mmol m^-^^3^/ 13 mg m^-^^3^) between surface-dominated and depth-dominated CaCO_3_ production regimes. Similar low surface PIC regimes (with annual surface PIC of <10 mg m^-^^3^) represent 87% of the surface of the ocean (Fig. S8); thus, assuming similar rates of CaCO_3_ production as the seasonally corrected production rates at Stations 1–4 globally puts a minimum estimate (assuming the remaining 13% of the ocean with higher surface PIC will have higher depth integrated production) for total global CaCO_3_ production of ~2.2×10^14^ mol yr^-1^ (2.6 Pg C yr^-1^), that is 0.8×10^14^ mol yr^-1^ using the production rate at Station 1 and 3.6×10^14^ mol yr^-1^ using the production rate at Station 3.

To make a first-order approximation of the impact of deepening CaCO_3_ production on global CaCO_3_ production we use a simple linear regression of total CaCO_3_ production at our sites against satellite PIC (CaCO_3_) (Fig. S2), with the deepening of production primarily manifesting as a non-zero intercept (note the high variability in the intercept coming from the changing production regimes from Stations 1 to 4 despite the low surface PIC at all Stations). We then apply this relationship to global satellite PIC climatology (Fig. S8). We include the caveat that 1) this assumes the bias caused by the deepening of CaCO_3_ production in the subtropics scales with surface PIC in a similar way globally^38^, and 2) the regression in Fig. S2 is driven by one station with high surface PIC (Station 5). While crude, this approach allows us to make a first-order approximation of the impact of deepening CaCO_3_ production on global CaCO_3_ production. Applying the relationship between total production and satellite PIC (Table S7) to the global mean surface satellite-derived PIC climatology (Fig. S8), and integrating globally (weighting by area) results in a total CaCO_3_ production of 3.1×10^14^ mol yr^-1^ (3.7 Pg C yr^-1^) globally. This estimate is similar to, although toward the upper end, of previous estimates of total pelagic calcification based on satellites, upper water column measurements, seasonal alkalinity changes, and ecosystem modeling which range from 0.9–3.9×10^14^ mol CaCO_3_ yr^-1^ (1.1–4.7 Pg C yr^-1^)^10,12,13,28,32,55^. However, as previously noted by others^10,13^ this estimate is considerably higher than estimates of global mean CaCO_3_ export flux from the upper ocean which ranges from 0.5–0.6×10^14^ mol yr^-1^ (0.6–0.7 Pg C yr^-1^; refs. ^7,8^).

While our estimate of the total amount of CaCO_3_ produced agrees well with that of Buitenhuis et al.^13^, there is a large discrepancy between our results and those of Buitenhuis et al.^13^ in terms of the dominant CaCO_3_ polymorph produced. We find CaCO_3_ production is dominated by calcitic coccolithophores, however, their results suggested pelagic CaCO_3_ production is mainly driven by aragonite pteropods with coccolithophores and foraminifera playing a minor role.

Given the discrepancy with the results of Buitenhuis et al.^13^ and the limited temporal interval of our sampling and the potential for large temporal variability of pteropod abundances^43^, we also calculate pteropod CaCO_3_ biomass and production in the North Pacific using the comprehensive MAREDAT database^18^, which has excellent spatial and seasonal sampling distribution in the North Pacific (Methods, Fig. 4). This results in a typical pteropod CaCO_3_ biomass in the upper 250 m of the North Pacific of 0.5 mg m^-3^ (0.2–1, 32–68% CI; note the dataset is highly skewed, Fig. S6), a vertically integrated pteropod CaCO_3_ biomass of 122 mg m^-2^ (50–269, 32–68% CI), and a pteropod CaCO_3_ production rate of 12 mg m^-2^ day^-1^ (5–27, 32–68% CI). The results calculated using the MAREDAT database are thus in good agreement with the estimates calculated using the samples collected during our own cruise (Fig. 3). We propose the discrepancy with the results of Buitenhuis et al.^13^ instead comes from three other factors: Firstly, their model parametrization uses a fixed PIC/POC ratio of 0.1 ref. ^56^ for coccolithophores; this value is substantially lower than the published review by Gafar et al.^25^ which ranged from 0.19 to 2.30 and much lower than the value 0.52 they used for pteropods, which is itself about two times higher than the estimates of Bednaršek et al.^18^ (Table 1). Secondly, they assume a similar turnover time for (single-celled) coccolithophores and (complex) pteropods, contrary to the available estimates from the literature^57–60^ (Table 1). Finally, within their calculation they assume all CaCO_3_ dissolving above the calcite saturation horizon is aragonite, an assumption which is likely to exaggerate aragonite production; as we discuss below, previous studies^52,61,27^ and our results indicate substantial dissolution of coccolithophore calcite above the calcite saturation horizon (which we attribute to respiration-driven dissolution and dissolution within the guts of grazers) such that this assumption is likely to be invalid.

**Table 1:** Ratio of Particular Inorganic carbon (PIC) to Particular Organic Carbon (POC) and turnover time (life span) for the calcifying taxon

Group	PIC:POC ratio	PIC:POC references	Turnover time (days)	Turnover time references
Coccolithophores	0.19–2.08^a^	ref. ^98^ and references therein	0.6–10 days (0.1–1.5 cell divisions per day)	ref. ^59^ and references therein
Pteropods	0.20–0.56^b^	ref. ^18,99^	5–16	ref. ^13,58,100^
Heteropods	0.28–0.45	This study	5–16	ref. ^13,58,100^
Planktonic foraminifera	3–6	ref. ^101^ and references therein; ref. ^102,103^	14–28^c^	ref. ^78^ and references therein

^a^C. leptoporus, E. huxleyi, C. pelagicus subsp. braarudii, G. oceanica, S. apsteinii, H. carteri, S. pulchra, U. sibogae.

^b^Limaciniidae and Cavoliniidae families.

^c^excludes deeper dwelling species with longer turnover times, however, these comprise only a very minor component of the assemblages^30,31^.

### CaCO_3_ sinking and export fluxes versus production

**Fig. 5:**
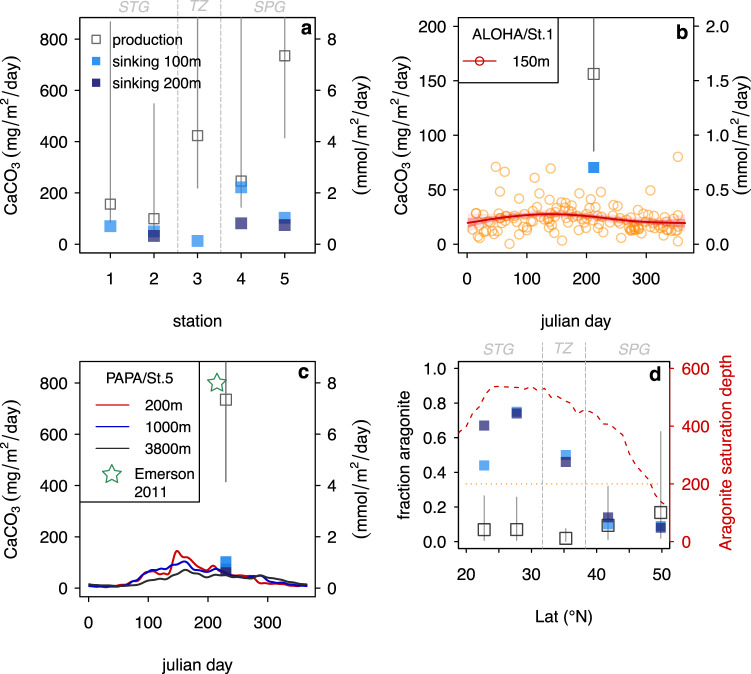
Pelagic CaCO_3_ production versus sinking fluxes. **a** total CaCO_3_ production versus sinking flux in the floating traps deployed at 100 m and 200 m during the plankton sampling at all stations (PIC concentrations not available at 200 m depth for stations 1 and 3)^34^ **b** Station 1/ALOHA ^65^ **c** Station 5/PAPA;^64^ turquoise star represents production estimate at PAPA from ref. ^54^ based on the seasonally cycle of in-situ pH and fCO_2_ **d** fraction aragonite in production and sinking flux in the floating traps deployed during the plankton sampling as a function of latitude; red dashed line shows the depth of aragonite saturation horizon (calculated from GLODAPv2^105^ and orange dotted line shows depth of deepest floating trap. Production in all panels is produced during the time of sampling (August 2017) i.e. it is not corrected for seasonal bias. Error bars for the total production (**a**, **b**, **c**) and fraction aragonite of production (**d**) represent the 95% CI (Methods) See legend in panel a for square symbols in panels (**c**, **d**, **e**). STG, TZ, and SPG represent subtropical gyre, transition zone, and subpolar gyre, respectively.

Our estimate of 3.1×10^14^ mol CaCO_3_ yr^-1^ global pelagic CaCO_3_ production is ~30 – 300 times larger than required to meet the 0.8 10^12^ - 1.1×10^13^mol CaCO_3_ buried in deep sea sediments each year^8,11,62–64^ and balance the riverine of input of alkalinity to maintain steady-state, reaffirming previous findings that most of the CaCO_3_ produced in the surface ocean is dissolved and recycled within the ocean interior (e.g., ref. ^8^). More surprisingly, at several stations our estimates of CaCO_3_ production are larger than the export fluxes at 100 to 200 m water depth in floating sediment trap deployed during the plankton sampling^34^, and our production estimates at Stations 1 and 5 are higher than the long running shallow sediment traps at Station ALOHA^65^ and Ocean Station PAPA^64,66,67^) (Fig. 5). While the discrepancy observed with the floating traps deployed during the sampling interval (in place for ~72 hrs) may be explained by a decoupling of CaCO_3_ production and natural mortality/sinking of pteropods, and coccolith aggregation, such that there could be a time lag between production at the surface and export through the water column, this time lag cannot explain the discrepancy observed with the long running sediment traps at Ocean Station PAPA and Station ALOHA. Our results show an annual production of 0.4 (0.2-2.1, 95% CI) and 0.9 (0.5-3.8) mol CaCO_3_ m^-2^ yr^-1^ at Stations 1 and 5, which is ~5 times higher than the annual export of 0.08 and 0.16 mol CaCO_3_ m^-2^ yr^-1^ at 150 m depth at ALOHA and 200 m at PAPA (Fig. 5c, d). We reiterate that previous estimates of annual CaCO_3_ production at PAPA station based on seasonal cycle of seawater carbonate chemistry support our production value^12,54,57^.

This disparity between the amount of CaCO_3_ produced, and the amount of CaCO_3_ that is exported out of the photic zone, suggests that a large portion (~80%) of the total CaCO_3_ produced in the photic zone is never exported, and is instead remineralised in situ; that is, only ~20% of the total CaCO_3_ produced is exported from the photic zone. Bishop & Wood^52^ suggested up to 92% of the total CaCO_3_ produced dissolved within the upper 500 m in the subpolar North Pacific. In situ remineralisation of such a high fraction of the CaCO_3_ that is produced within the photic zone explains the previous discrepancy between higher estimates of global CaCO_3_ production based on satellites, upper water column measurements, seasonal alkalinity changes, and ecosystem modeling (which all estimate the total amount of CaCO_3_ produced)^10,13,28^ and lower estimates of CaCO_3_ based on export production (such as sediment traps below the photic zone)^7,8^.

Shallow remineralisation of pteropods is suggested by the fraction of aragonite present in the shallow traps versus the production ratio, with the fraction of aragonite in the shallow traps decreasing northwards as the aragonite saturation horizon shoals, while the fraction produced increases (Fig. 4e); the highest fraction aragonite produced is at Station 5, where we also observe the lowest fraction in the shallow traps, and the aragonite saturation horizon shoals to above the depth of the shallow trap (<200 m). This dissolution pattern in living pteropod communities in the Gulf of Alaska has been observed recently by Bednaršek et al.^45^ However, our results indicate that not only aragonitic pteropod and heteropods dissolve^13^, but also a large amount of coccolithophore calcite. Our findings thus require CaCO_3_ dissolution above the calcite/aragonite saturation horizon throughout the North Pacific^27^. Tracers of excess alkalinity support widespread shallow dissolution, far above the calcite and aragonite saturation horizons, throughout the global ocean^23,27^.

The large amount of CaCO_3_ dissolution above the calcite/aragonite saturation horizon may be driven by multiple mechanisms, including localized undersaturation within the microenvironment driven by the remineralisation of organic matter, as well as dissolution within the guts of grazers and predators^23,33,45,68,69^. Both pteropods and coccolithophores contain a large fraction of organic carbon (Table 1), the respiration of which after their death can promote dissolution from the inside out (as well as making them a more attractive food source to grazers). Although the degree to which this could drive total dissolution of whole pteropod shells is still uncertain^70^, widespread dissolution of coccolithophore CaCO_3_ within the upper water column has been observed during bloom events (e.g., ref. ^71^). Furthermore, coccolithophores (which constitute by far the largest fraction of CaCO_3_ produced) disintegrate into individual coccoliths after death (this can be seen in the coccolith standing stocks in Fig. 2), and must sink by forming aggregates (e.g. marine snow). In situ micro-electrode measurements demonstrate a large pH drop with marine-snow during respiration^72^, which would drive further micro-environmental undersaturation and CaCO_3_ dissolution^27^. In addition, shallow sediment traps indicate coupling of PIC and POC remineralisation from 100 m to 200 m depth in the North Pacific^34^.

Although the processes by which coccolithophore CaCO_3_ dissolves in supersaturated waters remain uncertain, some insight may be gained from the residence time of loose coccolith CaCO_3_ within the production layer; dividing the loose coccolith CaCO_3_ standing stocks (Fig. 2) by the estimated whole coccosphere CaCO_3_ production rate at each station gives an approximate coccolith CaCO_3_ residence time on the order of several days to a couple of weeks. Given the requirement of coccoliths to sink by aggregation and fecal pellets^73^, the export and dissolution of coccolithophore calcite may occur in discrete events, possibly associated with episodes of high organic carbon production and grazing, rather than sinking as a steady rain, in agreement with previous suggestions based on seasonal export production in sediment trap studies (e.g., refs. ^4,50^). While our results suggest that a large portion of coccolithophore CaCO_3_ is remineralised in the photic zone, the very high rate of production means a substantial amount is still exported^4,5^, and incorporated within sediments^14^.

Foraminifera comprise ~20% of the total CaCO_3_ flux in a sediment trap at 3800 m at Station PAPA;^74^ however, our results show they consist of only ~4% of the total annual CaCO_3_ standing stock, and ~1% of the total annual CaCO_3_ production at Station 5. Due to their mass and low organic carbon content^24,75^ (Table 1), foraminifera sink quickly after death^50^, have a low self-dissolution potential, and are thus efficient exporters of CaCO_3_ out of the photic zone. Thunell & Honjo^74^ found the August flux of foraminiferal calcite in a sediment trap at 3800 m at Station PAPA ranged from 2−6 mg m^-2^ day^-1^. Our foraminiferal calcite production estimate at Station 5 is 9 mg m^-2^ day^-1^, suggesting foraminiferal calcite is efficiently exported into the deep ocean. Foraminifera thus play an important role in CaCO_3_ export and sedimentation, despite their low contribution to total production; estimates of CaCO_3_ in sediments suggest a ~50/50 ratio of coccolithophores/foraminifera^13^ versus the ~90/2 production ratio we observe.

While much attention has been given to decreasing calcification under ocean acidification^19,76^ our results indicate a decoupling of CaCO_3_ production and export (Fig. 5). As such, future changes in the processes driving shallow dissolution, and the ability to export CaCO_3_ out of the photic zone may play an equally important role in determining the future response of the CaCO_3_ cycle and its effects on the marine carbon cycle under anthropogenic climate change. Changes in grazing, particle aggregation, the PIC/POC ratio of the aggregates, or the relative abundance of foraminifera to coccolithophores/pteropods, could lead to large changes in the amount of CaCO_3_ exported from the surface ocean and thus the cycle of alkalinity. The PIC/POC ratio of coccolithophores has been demonstrated to decrease with increasing CO_2_ (ref. ^25^); if the dissolution of coccolithophore CaCO_3_ within the photic zone is in part related to the degree of calcification and/or the remineralisation of organic carbon contained within the soft tissue of the calcifying organisms, this decrease in the PIC/POC of coccolithophores may lead to a negative feedback with CO_2_, with increased dissolution (and thus reduced export of alkalinity) out of the surface ocean acting to buffer rising atmospheric CO_2_. Given the potential importance of CaCO_3_ export in driving changes in alkalinity and atmospheric CO_2_, and the large uncertainties in our current understanding, future work should focus on understanding the processes by which CaCO_3_ is either dissolved within the photic zone or exported to depth.

## Methods

### Sample collection

Samples were collected along a transect from Hawaii to Alaska during August 2017 as part of the CDisK-IV (KM1712) cruise on R/V Kilo Moana (Fig. 1). The five stations along the transect were designed to sample subtropical, transition zone, and subpolar waters. A rosette of Niskin bottles equipped with CTD (conductivity, temperature, depth) and other sensors for coccolithophore and biogeochemical parameters and a vertically integrated plankton tow were collected at each station. Further plankton tows were conducted at four additional intermediate stations (Supplementary material).

A 0.5 m diameter net with 90 µm mesh size was used throughout; based on previous work this mesh size should provide a good estimate of both pteropod^18,77^ and foraminiferal^78^ biomass. The sampling strategy was designed to capture an integrated sample of all foraminifera, pteropods, and heteropods from juveniles to adults living throughout the upper water column. The net was towed from the surface down to a specified maximum depth within the water column, and then back to the surface in a continuous manner following an oblique trajectory through the water column. The maximum depth was determined from the fluorescence profile of the preceding CTD cast, and was selected to ensure the net sampling captured well below the base of the chlorophyll maximum and ranged from 150 m in the most northerly subpolar sites to 300 m in the subtropical region (Tables S1, S2). The volume of water represented by each net tow sample was calculated by multiplying the net area by the distance traveled as determined by a flowmeter. For the vertically integrated values, the integration is carried out from the surface to the maximum depth of the tow.

After collection, samples were preserved in a 4% formalin seawater solution, buffered to a pH of ~8.1 with hexamethylenetetramine^73^. Samples were split with a Folsom splitter or a McLane rotary splitter (splitting error <4%). Large pteropods and heteropods (>1 mm) were picked and quantified before splitting. Half of the split sample was transferred into ethanol solution in the laboratory for the analysis of pteropods and heteropods.

Water samples from rosettes of Niskin bottles equipped with CTD (Sea-Bird SBE 9) were collected at different depths throughout the photic zone and including the chlorophyll maximum depth.

### Quantification of calcifying plankton community standing stock and biomass

All foraminifera were wet picked from the sample splits, divided into groups greater and less than 125 µm, counted, and weighed with a high precision microbalance. We assume the mass of organic matter is negligible since dry cytoplasm has no statistically significant effect on the weight of tests > 150 μm^24,75^. Empty tests made up a negligible component of the standing stock, typically comprising <2% of the total standing stock. Replicate picking and weighing of splits gave a typical reproducibility of ±4% (1σ). Foraminiferal assemblages from these samples were previously reported^31^.

Pteropods and heteropods were quantified and shell diameter was measured using a Leica Z16 AP0 binocular light microscope at 20−100×. Pteropods and heteropods were identified and grouped respectively in three (Cavoliniidae, Cymbuliidae, Limacinidae) and two (Atlantidae, Carinidae) families.

CaCO_3_ biomass (B) was estimated as follows: CaCO_3_ (mg) = PIC (mg) ×8.33 (assuming that all inorganic carbon is in the form of CaCO_3_), where the constant 8.33 represents the molecular mass ratio of carbon to CaCO_3_ and PIC is the Particulate Inorganic Carbon^79^. To estimate PIC we used the PIC/POC ratio of 0.27:0.73 calculated on pteropods by Bednaršek et al.^18^, where POC is the Particulate Organic Carbon (POC) representing the soft tissue of the organisms. POC was estimated by converting Wet Weight (WW, mg) to Dry Weight (DW, mg) using^80^ equation DW = WW × 0.28. DW was subsequently transformed to POC using the conversion factor POC = DW × 0.25 following^81^. WW and/or DW were calculated from the shell length (L, mm) using equations based on the different shell shape: ref. ^80,82,83^.

1 $${{{{{\rm{Cavoliniidae}}}}}},\,{{{{{\rm{WW}}}}}}=0.2152\times {{{{{{\rm{L}}}}}}}^{2.293},$$

2 $${{{{{\rm{Carinidae}}}}}},\,{{{{{\rm{WW}}}}}}=0.0888\times {{{{{{\rm{L}}}}}}}^{2.161},$$

3 $${{{{{\rm{Limacinidae}}}}}},\,{{{{{\rm{DW}}}}}}=0.1365\times {{{{{{\rm{L}}}}}}}^{1.501},$$

For Cymbulidae, we generated the following equation DW = (0.0392 × L)−0.003 from the measurement of shell (mm) and DW of 67 individuals.

For Atlantidae we generated the following equation CaCO_3_ = 0.769e^0,0023L^ (R^2^ = 0.885) from the measurement of shell length (mm) and CaCO_3_ biomass (mg) of 85 individuals. For the measurement of CaCO_3_ biomass heteropods were heated to 550 °C for 5 h to eliminate organic matter content and the ashes (representing the remains of the shells) weighed using a Toledo microbalance. The ash weight can be considered an indirect estimate of CaCO_3_ content^84^.

Between 2.1 and 6.0 liters of seawater were filtered onto Millipore cellulose acetate membranes, with 0.45 µm of pore size and 47 mm of diameter, for coccolithophore analysis. Filters were oven dried at 40 °C and stored in plastic petri dishes. A portion of each filter was mounted on a glass slide and analyzed by a polarized LEICA DM6000 light microscope at 1000× magnification along radial transects whose area was between 0.35 and 3.14 mm^2^. Cell concentrations per liter of seawater were estimated as follows:

4 $${{{{{\rm{Coccolithophore}}}}}}\,{{{{{\rm{concentration}}}}}}\,({{{{{\rm{number}}}}}}/{{{{{\rm{L}}}}}})=({{{{{\rm{F}}}}}}\times {{{{{\rm{C}}}}}})/({{{{{\rm{A}}}}}}\times {{{{{\rm{V}}}}}})$$

where F is the effective filtration area (mm^2^), C is the number of coccospheres, V is the filtered seawater volume (L) and A is the investigated filter area (mm^2^).

Taxa were identified following taxonomic concepts for living coccolithophores by ref. ^85,86^. To estimate the CaCO_3_ contribution by coccolithophore assemblages in each sample, we carried out the transformation of coccospheres in number of coccoliths, following the estimates by ref. ^87^, and then we adopted the coccolith mass estimates by^88^. The coccolith mass of Noëlaerhabdaceae also took into account the estimates by ref. ^89^ considering different mass for *E. huxleyi* calcification degree.

The estimate of coccosphere calcite was further complemented by the individual coccolith (detached from the coccosphere) calcite concentration quantified by microscopy along radial transects of 0.32 mm^2^, and subsequent mass estimates as described above.

The integrated total living coccolithophore calcite standing stock was obtained considering the first shallow sampling depth to a depth equal to 1% of the fluorescence peak. In St1 it ranges from 6 m to 180 m, Station 2 5–215 m, Staction 3 5–135 m; Staction 4 5–130 m, Staction 5: 5–130 m. To estimate the total annual coccolithophore calcite production we consider only the coccosphere calcite (number of calcifying cells). Uncertainty in the coccolith CaCO_3_ standing stock estimates is typically ±9% (1σ).

### From concentration to annual production

We converted the measured CaCO_3_ concentrations (i.e. CaCO_3_ standing stock, CaCO_3_ biomass) into production rate, using estimates of the turnover time for each group (that is, the typical lifespan of an individual; Table 1): for foraminifera we used a range of 10–30 days^90–93^, noting that more slowly reproducing deep-dwelling species make up only a very small fraction of the assemblages in our tows^31^. For pteropods and heteropods we used a range of 5–16 days (although we note their lifespan may be much longer than this^94^). Pteropods and heteropod turnover time was calculated as turnover time (days) =1/G, where G is the average instantaneous growth rates expressed as mg Ca deposited (on mg Ca shell)^−1^ day^−1^ ref. ^57,58^. We assume that growth rates do not vary with shell size; this approximation is supported by a previous study^77^, who found no significant difference in the shell growth rates of small and large sizes of any of the four pteropod species the author examined.

For coccolithophores we used a range of 0.1–1.5 cell division day^−1^ (1.5–10 days) (Table 1). This range is derived from laboratory field estimates and simulated by a generalized coccolithophore model for equatorial to North Pacific Ocean^59^. We are aware that cell growth phase differs for small cells with few coccoliths produced during exponential growth phase (normal, rapid division) and larger cells with more coccoliths produced during early stationary phase (slowed cell division).

Given the large range in the turnover rate of coccolithophores, foraminifera, pteropoda, and heteropoda, we apply a probabilistic approach to determine the production rate and propagate the uncertainties in turnover time through to our estimates of total production using a flat probability distribution i.e. for foraminifera there is equal chance of the average lifespan being 10 days as it is 30 days (this highly conservative approach thus results in larger total uncertainties in production rate). The production (mg m^-2^ day^-1^) is then given as the CaCO_3_ standing stock (in mg m^-^^2^) divided by the turnover time (days),

5 $${{{{{{\rm{CaCO}}}}}}}_{3}{{{{{\rm{production}}}}}}\,({{{{{\rm{mg}}}}}}\,{{{{{{\rm{m}}}}}}}^{-2}{{{{{{\rm{day}}}}}}}^{-1})\\ ={{{{{{\rm{CaCO}}}}}}}_{3}{{{{{\rm{standing}}}}}}\,{{{{{\rm{stock}}}}}}({{{{{\rm{mg}}}}}}\,{{{{{{\rm{m}}}}}}}^{-2})/{{{{{\rm{turnover}}}}}}\,{{{{{\rm{time}}}}}}({{{{{\rm{days}}}}}})$$

Our approach assumes that all of the organisms we sampled are living. This assumption is valid for foraminifera and pteropods as they sink individually, and relatively quickly upon death. For coccolithophores this assumption is valid as we only consider intact coccospheres, which mostly disaggregate quickly upon death. Annual estimates were then calculated by multiplying the daily estimates by 365 accounting for the seasonal bias at the time of sampling using PIC/chlorophyll_a/zooplankton time series (see below).

The data and R code to perform the calculation of CaCO_3_ production including error propagation and seasonal bias correction (see below) is available at 10.5281/zenodo.7458132.

### Correction of production for seasonality and interannual variability

To account for seasonal/inter-annual bias (or specifically, the bias at the time of sampling compared to mean annual production) we use satellite-derived PIC (CaCO_3_) to correct the coccolithophore production estimates (Fig. S7). The rationale behind this is that although satellites only capture coccolithophore PIC concentrations in the upper few meters of the water column, the relative seasonal/inter-annual changes at the surface should broadly reflect the relative depth integrated seasonal/inter-annual changes in production at depth^10,38^. We assess the validity of using satellite-derived PIC by regressing satellite-derived PIC (CaCO_3_) estimates during August 2017 against the surface values of coccolithophore CaCO_3_ (Fig. S1). Our surface (~5 m depth) estimates of coccolithophore CaCO_3_ standing stock show a strong correlation with satellite PIC (CaCO_3_) during August 2017. For each site the seasonal bias factor is calculated as satellite PIC during August 2017)/satellite mean annual PIC (2009–2019). Annual mean coccolithophore production is then given as depth integrated coccolithophore CaCO_3_ production during August 2017 × 1/seasonal bias factor. As a sensitivity experiment, we repeated this exercise using satellite-derived chlorophyll (see below) instead of PIC, which results in larger estimates of annual CaCO_3_ production than using PIC.

Unlike coccolithophores, we have no way to directly measure changes in foraminiferal CaCO_3_ production through time. Instead, we use satellite-derived Chlorophyll A (chlor_a) to correct the foraminiferal production estimates for seasonal/interannual changes (Fig. S9). The rationale here is that the seasonal flux of foraminifera in the North Pacific has been shown to follow primary production^31,74^, such that we can use relative changes in chlorophyll through time at each site to correct the foraminiferal production estimates. For each site the seasonal bias factor is calculated as chlor_a during August 2017)/mean annual chlor_a (2002–2019). Annual mean foraminiferal production is then given as foraminiferal CaCO_3_ production during August 2017 × 1/seasonal bias factor.

Again, as we have no way to directly measure changes in pteropod/heteropod CaCO_3_ production through time, for heteropods and pteropods we refer to the long-term zooplankton data set from ocean stations ALOHA and PAPA to correct pteropod and heteropod CaCO_3_ production for seasonality. The rational here is that the seasonal changes in pteropod/heteropod abundance should broadly follow the seasonal changes in zooplankton abundance^57^. We note, that unlike the satellite PIC and chlorophyll estimates used for coccolithophores and foraminifera, this method is not able to account for interannual variability, and only adjusts for the seasonal trend. Based on the multidecadal data set of total zooplankton biomass at St. PAPA^66,67^ and St. ALOHA (all data and metadata are publicly available at hahana.soest.hawaii.edu/hot/hot-dogs/interface.html) the mean zooplankton biomass in the summer is respectively 2 and 1.2 times greater than the mean annual zooplankton biomass. We extrapolate these values of seasonal bias to each of our sites using latitude. We assume undetectable seasonal variation in pteropod growth rates. If growth rates of pteropods could slightly decrease with temperature, the annual production of aragonite would be less. Given the large assumptions within our method of correcting the pteropod and heteropod production data for seasonal variability, and the possibility of large temporal variability in pteropod abundances^43^, we also calculate annual pteropod CaCO_3_ production using the comprehensive pteropod biomass compilation of Bednaršek et al.^18^ (see below), which has excellent spatial and temporal sampling in the North Pacific (below).

### Pteropod biomass and production estimated from MAREDAT

Given the discrepancy observed with previous estimates of pteropod CaCO_3_ production^13^ and the sparse spatial and temporal resolution of our net-tow sampling, which unlike the coccolithophore data, cannot be independently verified using satellite data, we also estimate pteropod CaCO_3_ biomass and production in the North Pacific using the MAREDAT database^17^. The database has excellent spatial and temporal coverage of pteropod sampling within the North Pacific (Fig. 5). Similar to Bednaršek et al.^18^, we find no significant trends in biomass by latitude or time of year, so we perform our analysis using all samples spanning the entire North Pacific basin and for all months. We take all estimates of pteropod carbon biomass (reported in the database as mg C m^-^^3^) from the upper 250 m of the water column, which we consider as the production layer, with the vast majority of pteropod biomass found within the upper 200m^18,95^. This results in 1793 discrete observations. We include zero values (120 samples, <7% total) and remove 3σ outliers (16 samples, <1% total). Including these outliers results in unstable Gaussian Kernal densities (see below). We calculate the CaCO_3_ biomass as the carbon biomass multiplied by the fraction PIC (*f*PIC), using PIC:POC estimates given in Table 1. We then integrate the standing stock over the production layer, and calculate the production by diving by the turnover time (Table 1) in the same manner as for the C-Disk-IV samples. We calculate the uncertainties via Bootstrapping of the dataset, propagating the uncertainty in PIC:POC and turnover time using Monte-Carlo simulation, using a highly-conservative flat probability distribution for both. Using a different depth range for the production layer (i.e. 0–1000 m) has a negligible effect on our results.

We estimate the probability of pteropod carbon and CaCO_3_ biomass in the upper 250 m, integrated CaCO_3_ biomass, and CaCO_3_ production rate using truncated kernal density:^96^ this approach truncates the densities below zero, up-weighting the values that are closest to zero, and thus deals better with the highly-skewed dataset containing zero values.

In the North Pacific we find a typical CaCO_3_ biomass in upper 250 m of 0.5 mg m^-3^ (0.2–1, 32–68% range), vertically integrated CaCO_3_ biomass in upper 250 m of 122 mg m^-2^ (50–269, 32–68% range), daily CaCO_3_ production of 12 mg m^-2^ day^-1^ (5–27, 32-68% range).

Expanding our analyzes the global dataset of ref. ^18^ (i.e. not only the North Pacific subset described above) we find the global dataset is heavily skewed (Skewness = 13.3; a value above 1 is considered Skewed), such that the mean value reported by ref. ^18^ is not a useful statistic to describe the dataset. Using the same kernal density method used above for the North Pacific subset we find a typical pteropod CaCO_3_ biomass of 0.02 mg m^-3^ globally using data from the upper 250 m, or 0.04 mg m^-3^, considering all data in the upper 1000 m (Fig. S6). These values are three orders of magnitude smaller than the mean pteropod CaCO_3_ biomass value reported by ref. ^18^.

### Global satellite-derived PIC

In order to extrapolate the implications of our CaCO_3_ production estimates globally, we utilize a global climatology of satellite PIC (CaCO_3_) (Fig. S8;^97^). Our results indicate satellite PIC (MODIS CaCO_3_) estimates in the subtropical gyre are biased low compared to vertically integrated CaCO_3_ production, due to the deeper (mainly coccolithophore) CaCO_3_ production depth and the thickness of the coccolithophore productive layer (Fig. 2). To account for this bias in the CaCO_3_ estimates we use a simple regression of total CaCO_3_ production at our sites against satellite CaCO_3_,

6 $${{{{{{\rm{annual}}}}}}}\;{{{{{\rm{production}}}}}}\,{{{{{{\rm{CaCO}}}}}}}_{3}\;({{{{{\rm{mol}}}}}}\,{{{{{{\rm{m}}}}}}}^{2}{{{{{{\rm{yr}}}}}}})={{{{{{\rm{0.65}}}}}}}\;+{{{{{\rm{0.03}}}}}}\, \\ \times {{{{{\rm{satellite}}}}}}\;{{{{{\rm{PIC}}}}}}\;({{{{{\rm{mg}}}}}}\,{{{{{{\rm{m}}}}}}}^{-3})$$

R^2^ = 0.71, *p* < 0.05, standard error = 0.3 mol m^2^ yr^-1^ (Fig. S2; Table S8). The deepening of production manifests as the non-zero intercept. Note this relationship is driven by one station with high surface PIC (Station 5). Applying the relationship between total production and satellite PIC (Fig. S2, Table S8) to the global mean surface satellite derived PIC climatology (Fig. S7, Table S8), and integrating globally (weighting by area) results in a total CaCO_3_ production of 3.1 10^14^ mol yr^-1^ (3.7 Pg C yr^-1^) globally, with the large caveat that this assumes the bias caused by the deepening of production away from the high latitudes scales with surface PIC similarly globally. While we acknowledge this approach is very crude, it nevertheless provides us with a first-order approximation of total global production of CaCO_3_ implied by our results. The MODIS PIC data are available from NASA Goddard Space Flight Center, Ocean Ecology Laboratory, Ocean Biology Processing Group^97^.

### Floating sediment trap deployments

The methods and data for the floating sediment traps are given in Dong et al.^34^ To summarize, at Stations 1–5 (Fig. 1) an array of surface-tethered sediment traps was deployed on a single line; one at 100 m and the other at 200 m depth. Traps were deployed as free-floating arrays for 52 to 78 h. The traps were polycarbonate particle interceptor tubes (PIT) that were 70 cm long, 10 cm diameter (12 tubes per trap) with funnels inserted to guide particles into a Falcon tube attached to the end of the funnel. Falcon tubes were pre-filled with HgCl_2_ poison in brine solutions to inhibit diffusive loss of poison during deployment. The poison-brine solution was made from seawater collected at 150 m with NaCl added to increase the salinity by ∼5, and sodium borate was added to increase alkalinity by ∼2000 μM (US JGOFS protocol). Samples from six arbitrarily-chosen tubes among the 12 tubes at the same depth were combined and ‘swimmers’ were manually picked out. The samples were then filtered onto a pre-weighed glass fiber filter (Whatman glass microfiber filters, Grade GF/F, 1825–047) and, after being returned to the lab, were reweighed to calculate sinking mass flux. The solid materials on the filters were then collected and analyzed with XRD for mineralogy (aragonite/calcite), and with the Picarro for PIC and total C. We refer the reader to Table 1 in ref. ^34^ for the values aragonite/calcite ratios and fluxes.

## Supplementary information

Supplementary Information

## Data Availability

The data are given in Tables S1-6 in Supplementary Information and are available on Pangaea (10.1594/PANGAEA.948508).

## Code availability

The data and R code to perform the calculation of CaCO_3_ production including error propagation and seasonal bias correction is available at 10.5281/zenodo.7458132.
