# Supplementary material for: Pelagic calcium carbonate production and shallow dissolution in the North Pacific Ocean
Source: Nat Commun. 2023 Feb 20;14:805. doi: 10.1038/s41467-023-36177-w (PMC9941586; doi:10.1038/s41467-023-36177-w)
Supplement: Supplementary file 1 — Supplementary Information [file 41467_2023_36177_MOESM1_ESM.pdf]

Pelagic calcium carbonate production and shallow dissolution in the North Pacific Ocean

Supplementary Information

Patrizia Ziveri[1,2,3*], William Robert Gray[4,5,*], Griselda Anglada-Ortiz[1,6], Clara Manno[7], Michael Grelaud[1], Alessandro Incarbona[8], James William Buchanan Rae[5], Adam V. Subhas[9], Sven Pallacks[1], Angelicque White[10], Jess F. Adkins[11], William Berelson[12]

[1]Universitat Autònoma de Barcelona, Institute of Environmental Science and Technology, Barcelona, Spain
[2]Catalan Institution for Research and Advanced Studies (ICREA), Barcelona, Spain
[3]Universitat Autònoma de Barcelona, BABVE Department, Barcelona, Spain
[4]Laboratoire des Sciences du Climat et de l'Environnement (LSCE/IPSL), Université Paris-Saclay, Gif-sur-Yvette, France
[5]University of St Andrews, School of Earth and Environmental Sciences, St Andrews, United Kingdom
[6]Centre for Arctic Gas Hydrate, Environment and Climate (CAGE), Department of Geosciences, UiT The Arctic University of Norway, Tromsø, Norway
[7]British Antarctic Survey, Natural Environmental Research Council, Cambridge, United Kingdom
[8]Università di Palermo, Dipartimento di Scienze della Terra e del Mare, Palermo, Italy
[9]Department of Marine Chemistry and Geochemistry, Woods Hole Oceanographic Institution, Woods Hole, MA, United States
[10]School of Ocean and Earth Science and Technology, Department of Oceanography, University of Hawai‘i at Manoa, Honolulu, United States
[11]Department of Geology and Planetary Sciences, Linde Center for Global Environmental Science, California Institute of Technology, Pasadena, CA, United States
[12] University of Southern California, Department of Earth Sciences, Los Angeles, CA, United States

*these authors contributed equally to this work
Correspondence to: Patrizia.Ziveri@uab.cat and william.gray@lsce.ipsl.fr

The data from this study are available online in a machine readable format at https://doi.pangaea.de/10.1594/PANGAEA.948508.

Table S1a: Station locations for the CDisK-IV cruise CTD casts used for coccolithophore analysis. Station 1 was conducted at Station ALOHA/HOT (22°45’0.0” N, 158°0’0.0” W); Station 5 was conducted near ocean station PAPA (50°6’0.0” N, 144°54’0.0” W). Floating sediment traps sites were deployed near CTD stations.

**Station**	**Latitude**	**Longitude**	**Date**	**Starting Time**
1	22°45’14.4” N	157°58’40.8” W	1/08/2017	19:07
2	27°43’58.8” N	155°15’0.0” W	5/08/2017	23:31
3	35°16’1.2” N	150°58’58.8” W	13/08/2017	02:56
4	41°45’0.0” N	148°15’0.0” W	16/08/2017	22:50
5	49°49’58.8” N	149°13’1.2” W	21/08/2017	11:12

Table S1b: Station locations for the CDisK-IV cruise oblique plankton tow sampling. Stations where coccolithophore biomass was measured are indicated with *. Station 1 was conducted at Station ALOHA/HOT (22°45’0.0” N, 158°0’0.0” W); Station 5 was conducted near ocean station PAPA (50°6’0.0” N, 144°54’0.0” W).

**Station**	**Date**	**Time start**	**Time end**	**Lat start (°N)**	**Lat end (°N)**	**Lon start (°W)**	**Lon end (°W)**	**target depth (m)**	**Seawater volume (m3)**
1*	3/8/17	03:50	04:30	22°45.247'	22°45.191'	157°58.696'	157°58.753'	0-300	87.20
2*	6/8/17	09:26	10:11	27°44.451'	27°44.073'	155°15.196'	155°14.634'	0-300	142.49
2.5	11/8/17	17:55	18:29	31°30.644'	31°30.836'	153°29.526'	153°29.352'	0-300	74.06
3*	13/8/17	04:45	05:22	35°16.346'	35°16.810'	150°59.754'	150°59.522'	0-250	74.25
3.5	16/8/17	02:55	03:30	38°29.710'	38°30.145'	149°37.876'	149°37.716'	0-200	66.02
4*	17/8/17	03:12	03:30	41°45.714'	41°45.490'	148°15.697'	148°16.108'	0-200	44.69
4.5	20/8/17	10:53	11:32	45°54.011'	45°55.308'	148°53.696'	148°52.811'	0-150	313.13
5*	24/8/17	05:36	06:05	49°49.285'	49°50.77'	149°12.901'	149°13.612'	0-150	56.12
6.5	28/8/17	10:23	10:58	58°53.701'	58°54.533'	149°32.687'	149°31.715'	0-125	190.26

Table S2: $CaCO_3$ standing stocks (mg m^{-2}) of the different of planktic calcifying groups considered in this study, numbers in italic refer to the 1σ, numbers in parenthesis refer to $CaCO_3$ standing stocks in mmol m^{-2}.

Station	Pteropods		Heteropods		Foraminifers		Coccolithophores	
1	111.00<br>(1.11)	*15.65*	3.16<br>(0.03)	*0.45*	36.67<br>(0.37)	*1.47*	750.42<br>(7.50)	*67.54*
2	63.68<br>(0.64)	*8.98*	8.42<br>(0.08)	*1.19*	8.70<br>(0.09)	*0.35*	478.84<br>(4.78)	*43.10*
2.5	231.3<br>(2.31)	*32.6*	81.6<br>(0.82)	*11.5*	4.73<br>(0.05)	*0.19*	*No data*	
3	78.89<br>(0.79)	*11.12*	4.17<br>(0.04)	*0.59*	1.38<br>(0.01)	*0.06*	2173.28<br>(21.71)	*195.60*
3.5	70.72<br>(0.71)	*9.97*	2.64<br>(0.03)	*0.37*	3.20<br>(0.03)	*0.13*	*No data*	
4	215.29<br>(2.15)	*30.36*	24.76<br>(0.25)	*3.49*	404.44<br>(4.04)	*16.18*	1047.05<br>(10.46)	*94.23*
4.5	147.75<br>(1.48)	*20.83*	0.00<br>(0.00)	*0.00*	1.19<br>(0.01)	*0.05*	*No data*	
5	1306.44<br>(13.05)	*184.21*	0.00<br>(0.00)	*0.00*	182.22<br>(1.82)	*7.29*	3045.58<br>(30.43)	*274.10*
6.5	93.45<br>0.93	*13.18*	0.00<br>(0.00)	*0.00*	3.75<br>(0.04)	*0.15*	*No data*	

Table S3: Living coccolithophores $CaCO_3$ standing stocks (mg m^{-3}). Numbers in parenthesis refer to $CaCO_3$ standing stocks in mmol m^{-3}.

Depth (m)	Station 1	Station 2	Station 3	Station 4	Station 5
5	-	4.170 (0.042)	4.188 (0.042)	15.837 (0.158)	44.565 (0.445)
6	1.860 (0.019)	-	-	-	-
9	-	-	8.161 (0.082)	-	-
10	-	-	-	-	26.717 (0.267)
20	-	-	-	21.737 (0.217)	60.478 (0.604)
23	-	-	12.365 (0.124)	-	-
30	6.845 (0.068)	2.628 (0.026)	-	17.717 (0.177)	110.621 (1.105)
50	3.409 (0.034)	0.032 (0.000)	6.604 (0.066)	9.051 (0.090)	19.756 (0.197)
75	3.436 (0.034)	1.442 (0.014)	48.043 (0.480)	3.513 (0.035)	0.000 (0.000)
100	10.989 (0.110)	1.533 (0.015)	17.764 (0.177)	3.034 (0.030)	0.000 (0.000)
120	4.402 (0.044)	9.287 (0.093)	2.832 (0.028)	-	-
125	-	-	-	0.938 (0.009)	0.171 (0.002)
150	-	2.094 (0.021)	1.130 (0.011)	0.013 (0.000)	0.172 (0.002)
175	0.134 (0.001)	0.177 (0.002)	-	-	-
200	0.009 (0.000)	0.007 (0.000)	-	-	-

Table S4: Detached coccolith $CaCO_3$ standing stocks (mg m^{-3}). Numbers in parenthesis refer to $CaCO_3$ standing stocks in mmol m^{-3}.

Depth (m)	Station 1	Station 2	Station 3	Station 4	Station 5
5	-	0.000 (0.000)	0.000 (0.000)	0.000 (0.000)	28.447 (0.284)
6	0.000 (0.000)	-	-	-	-
9	-	-	0.000 (0.000)	-	-
10	-	-	-	-	26.871 (0.268)
20	-	-	-	5.255 (0.053)	55.253 (0.552)
23	-	-	27.777 (0.278)	-	-
30	0.000 (0.000)	0.000 (0.000)	-	4.246 (0.042)	64.393 (0.643)
50	1.033 (0.010)	0.000 (0.000)	43.873 (0.438)	3.848 (0.038)	22.726 (0.227)
75	0.000 (0.000)	3.417 (0.034)	37.263 (0.372)	8.084 (0.081)	0.000 (0.000)
100	0.000 (0.000)	0.000 (0.000)	11.587 (0.116)	4.075 (0.041)	0.442 (0.004)
120	3.505 (0.035)	4.329 (0.043)	23.489 (0.235)	-	-
125	-	-	-	3.127 (0.031)	0.000 (0.000)
150	-	0.000 (0.000)	19.469 (0.195)	0.000 (0.000)	0.000 (0.000)
175	0.000 (0.000)	0.000 (0.000)	-	-	-
200	0.000 (0.000)	2.462 (0.025)	-	-	-

Table S5: Seasonally corrected annual $CaCO_3$ production (mg $CaCO_3$ m^{-2} yr^{-1}) of the different groups of planktic calcifiers considered in this study. Numbers in parenthesis refer to $CaCO_3$ production in mmol m^{-2} yr^{-1} and numbers in italic refer to lower and upper estimates for each group and sample. Uncorrected total production values presented in Table S7.

Station		Pteropods	Heteropods	Foraminifers	Coccolithophores	Calcite	Aragonite	Total
1	Estimate	3214.93 (32.12)	91.04 (0.91)	456.07 (4.56)	34337.41 (343.08)	34838.62 (348.08)	3316.08 (33.13)	38669.64 (386.36)
	Lower	*1845.90 (18.44)*	*52.51 (0.52)*	*302.16 (3.02)*	*17880.99 (178.65)*	*18383.56 (183.68)*	*1946.04 (19.44)*	*21735.37 (217.16)*
	Upper	*6800.73 (67.95)*	*192.53 (1.92)*	*865.64 (8.65)*	*203735.52 (2035.59)*	*204261.05 (2040.84)*	*6901.22 (68.95)*	*208798.15 (2086.17)*
2	Estimate	1640.15 (16.39)	217.52 (2.17)	99.37 (0.99)	20163.66 (201.46)	20276.81 (202.59)	1882.99 (18.81)	22396.91 (223.77)
	Lower	*949.54 (9.49)*	*126.11 (1.26)*	*65.74 (0.66)*	*10474.07 (104.65)*	*10583.89 (105.75)*	*1157.73 (11.57)*	*12458.02 (124.47)*
	Upper	*3482.53 (34.80)*	*455.80 (4.55)*	*188.05 (1.88)*	*118923.06 (1188.20)*	*119021.33 (1189.18)*	*3747.52 (37.44)*	*121254.12 (1211.49)*
3	Estimate	1758.73 (17.57)	92.52 (0.92)	14.73 (0.15)	85514.85 (854.41)	85533.96 (854.60)	1860.15 (18.59)	87676.44 (876.00)
	Lower	*996.85 (9.96)*	*53.06 (0.53)*	*9.75 (0.10)*	*44547.77 (445.09)*	*44558.82 (445.20)*	*1093.86 (10.93)*	*46718.30 (466.78)*
	Upper	*3705.76 (37.03)*	*194.82 (1.95)*	*27.88 (0.28)*	*500982.70 (5005.48)*	*500994.38 (5005.59)*	*3825.77 (38.22)*	*504621.20 (5041.83)*
4	Estimate	4261.75 (42.58)	488.51 (4.88)	8409.36 (84.02)	80424.07 (803.54)	89649.24 (895.71)	4812.21 (48.08)	95160.98 (950.78)
	Lower	*2483.24 (24.81)*	*280.08 (2.80)*	*5567.34 (55.63)*	*42097.33 (420.61)*	*50349.81 (503.06)*	*2965.22 (29.63)*	*55225.33 (551.77)*
	Upper	*9022.38 (90.15)*	*1029.52 (10.29)*	*15997.22 (158.83)*	*478909.88 (4784.94)*	*487745.88 (4873.22)*	*9558.64 (95.50)*	*493012.88 (4925.85)*
5	Estimate	22801.34 (227.82)	0.00 (0.00)	964.14 (9.63)	59475.17 (594.24)	60528.86 (604.76)	22801.34 (227.82)	87934.63 (878.58)
	Lower	*13052.48 (130.41)*	*0.00 (0.00)*	*639.74 (6.39)*	*31036.70 (310.10)*	*32048.34 (320.21)*	*13052.48 (130.41)*	*50917.99 (508.74)*
	Upper	*48154.64 (481.13)*	*0.00 (0.00)*	*1829.17 (18.28)*	*348520.60 (3482.18)*	*349260.93 (3489.58*	*48154.64 (481.13)*	*375499.60 (3751.74)*

Table S6: standing stocks (ind m^{-2} and ind m^{-3}) of the different planktic calcifying groups considered in this study. Numbers in italic refer to the 1σ.

Station		Pteropods		Heteropods		Foraminifers	
1	ind.m^{-2}	6101.8	*482.0*	2288.2	*180.8*	16652.9	*2164.9*
	ind.m^{-3}	22.02	*1.74*	8.26	*0.65*	60.09	*7.81*
2	ind.m^{-2}	12420.1	*981.2*	3579.3	*282.8*	13422.3	*1744.9*
	ind.m^{-3}	38.96	*3.08*	11.23	*0.89*	42.11	*5.47*
2.5	ind.m^{-2}	6275.2	*495.7*	3176.3	*250.9*	1781.8	*231.6*
	ind.m^{-3}	21.87	*1.73*	11.07	*0.87*	6.21	*0.81*
3	ind.m^{-2}	8006.3	*632.5*	1103.4	*87.2*	3502.8	*455.4*
	ind.m^{-3}	33.62	*2.66*	4.63	*0.37*	14.71	*1.91*
3.5	ind.m^{-2}	3985.4	*314.8*	2277.4	*179.9*	2135.0	*277.6*
	ind.m^{-3}	16.96	*1.34*	9.69	*0.77*	9.09	*1.18*
4	ind.m^{-2}	25571.4	*2020.1*	9421.0	*744.3*	189262.0	*24604.1*
	ind.m^{-3}	108.85	*8.60*	40.10	*3.17*	805.63	*104.73*
4.5	ind.m^{-2}	5933.7	*468.8*	0.0	*0.0*	274.0	*35.6*
	ind.m^{-3}	44.26	*3.50*	0.00	*0.00*	2.04	*0.27*
5	ind.m^{-2}	121686.0	*9613.2*	0.0	*0.0*	255730.7	*33245.0*
	ind.m^{-3}	802.92	*63.43*	0.00	*0.00*	1687.39	*219.36*
6.5	ind.m^{-2}	5817.3	*459.6*	0.0	*0.0*	792.0	*103.0*
	ind.m^{-3}	44.78	*3.54*	0.00	*0.00*	6.10	*0.79*

Table S7: Satellite-derived PIC for August 2017. The data were extracted using the "Pixel Extraction" tool from SeaDAS (version 7.5.3; https://seadas.gsfc.nasa.gov/). The coordinates used to extract the satellite-derived PIC data correspond to the exact location where the samples were collected. The data were extracted from a 3x3 pixels window centred on each station's location and averaged. Surface cocco CaCO3 is both the living coccolithophores and detached coccoliths $CaCO_3$ standing stocks. Total CaCO3 production is uncorrected for seasonal bias (i.e. production estimate at the time of sampling). These data are plotted in Figure S2.

Station	Latitude	Longitude	satellite-derived PIC (mol.m^{-3}) ***PIC (mg.m^{-3})***	Sigma PIC (mol.m^{-3}) ***Sigma PIC (mg.m^{-3})***	Surface cocco $CaCO_3$ (mg.m^{-3}) ***Surface cocco $CaCO_3$ (mmol.m^{-3})***	Total $CaCO_3$ production uncorrected for seasonal bias (mol m^{-2} yr^{-1} *95% CI range*
1	22.7574	-157.9798	0.04622 ***4.6260***	0.00474 ***0.4742***	1.860 ***0.01858***	0.6 *0.3-3.1*
2	27.7428	-153.2547	0.04978 ***4.9819***	0.00367 ***0.3670***	4.170 ***0.04166***	0.4 *0.2-2.0*
3	35.2652	-150.9985	0.06289 ***6.2941***	0.00686 ***0.6870***	4.188 ***0.04184***	1.5 *0.8-8.8*
4	41.7422	-148.2997	0.08844 ***8.8519***	0.00921 ***0.9215***	15.837 ***0.15823***	0.9 *0.5-4.5*
5	49.6701	-149.5841	0.68644 ***68.7039***	0.08349 ***8.3564***	73.012 ***0.72949***	2.66 *1.5-12.6*

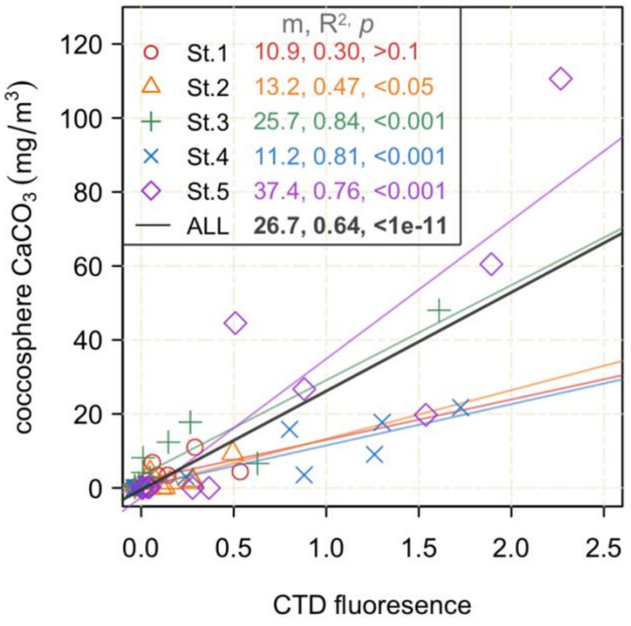

Supplementary Figure S1: Scatter plot of coccosphere $CaCO_3$ standing stock versus CTD fluorescence at the 5 stations. The colored lines show linear regressions with data from the individual stations, and the black line is a regression of data from all stations combined.

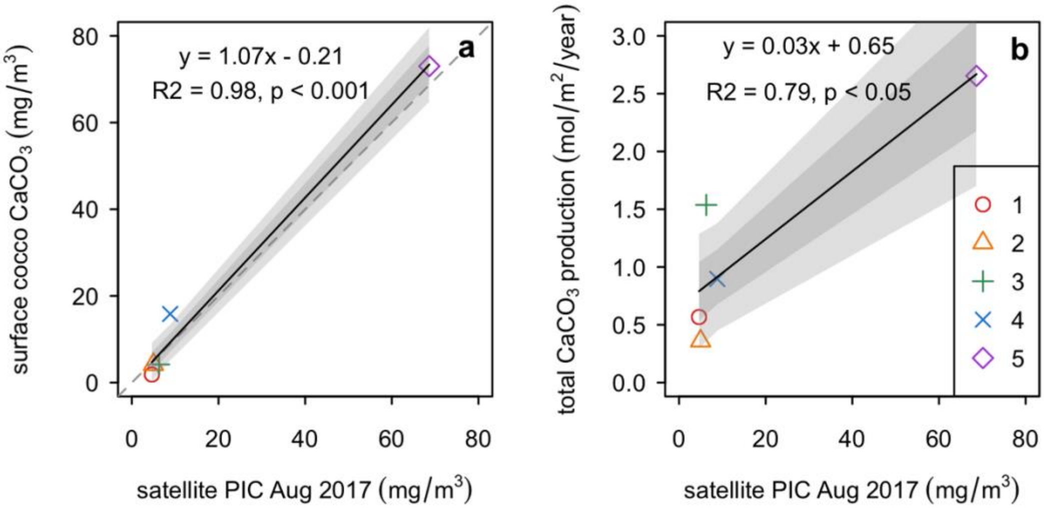

Supplementary Figure S2 (a) Standing stock of coccosphere and coccolith $CaCO_3$ at the first sampling depth (~5 m) and satellite PIC for August 2017 (mg $CaCO_3$ m^{-3}; Table S7); solid line and shaded area shows least squares regression, and the 1:1 line is shown by the dashed line. **(b)** total $CaCO_3$ production rate at the time of sampling (i.e. not corrected for seasonal bias) versus satellite PIC for August 2017; solid line and shaded area shows least squares regression (annual production $CaCO_3$ (mol m^2 yr) = 0.65 + 0.03*satellite PIC (mg m^3); R^2=0.71, p<0.05, standard error = 0.3 mol m^{-2} yr^{-1}). The data to reproduce this figure are given in Table S7.

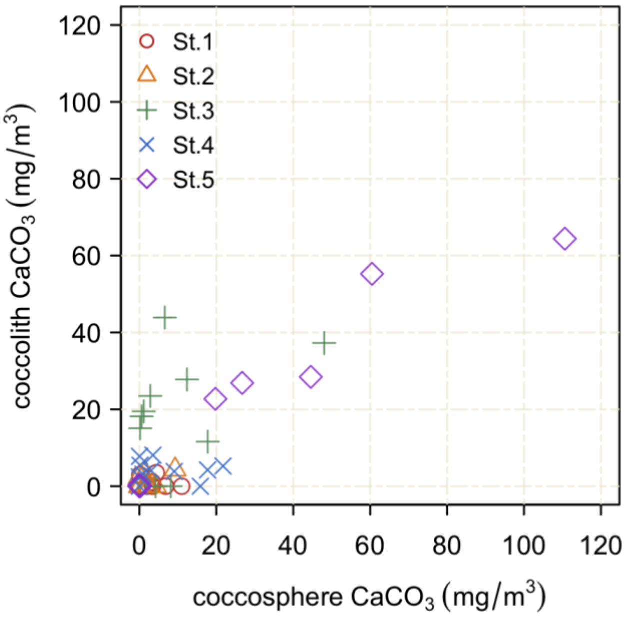

Supplementary Figure S3: Scatter plot of detached coccolith $CaCO_3$ and coccosphere $CaCO_3$ standing stocks in the 5 stations.

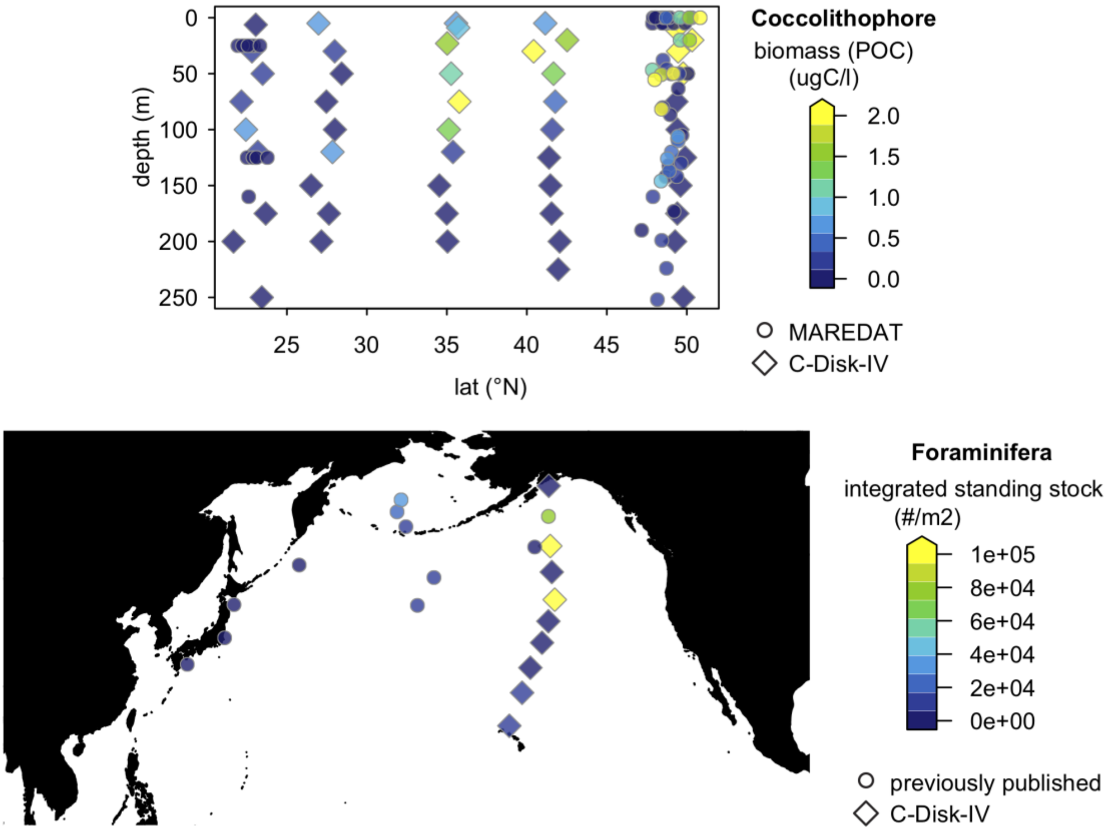

Supplementary Figure S4: (upper) comparison of coccolithophore biomass in C-Disk-IV to MAREDAT compilation (1, 2), in µg POC/l. **(lower)** comparison foraminiferal integrated biomass in C-Disk-IV samples to previously published data from the North Pacific, in #individual foraminifera m^{-2}.

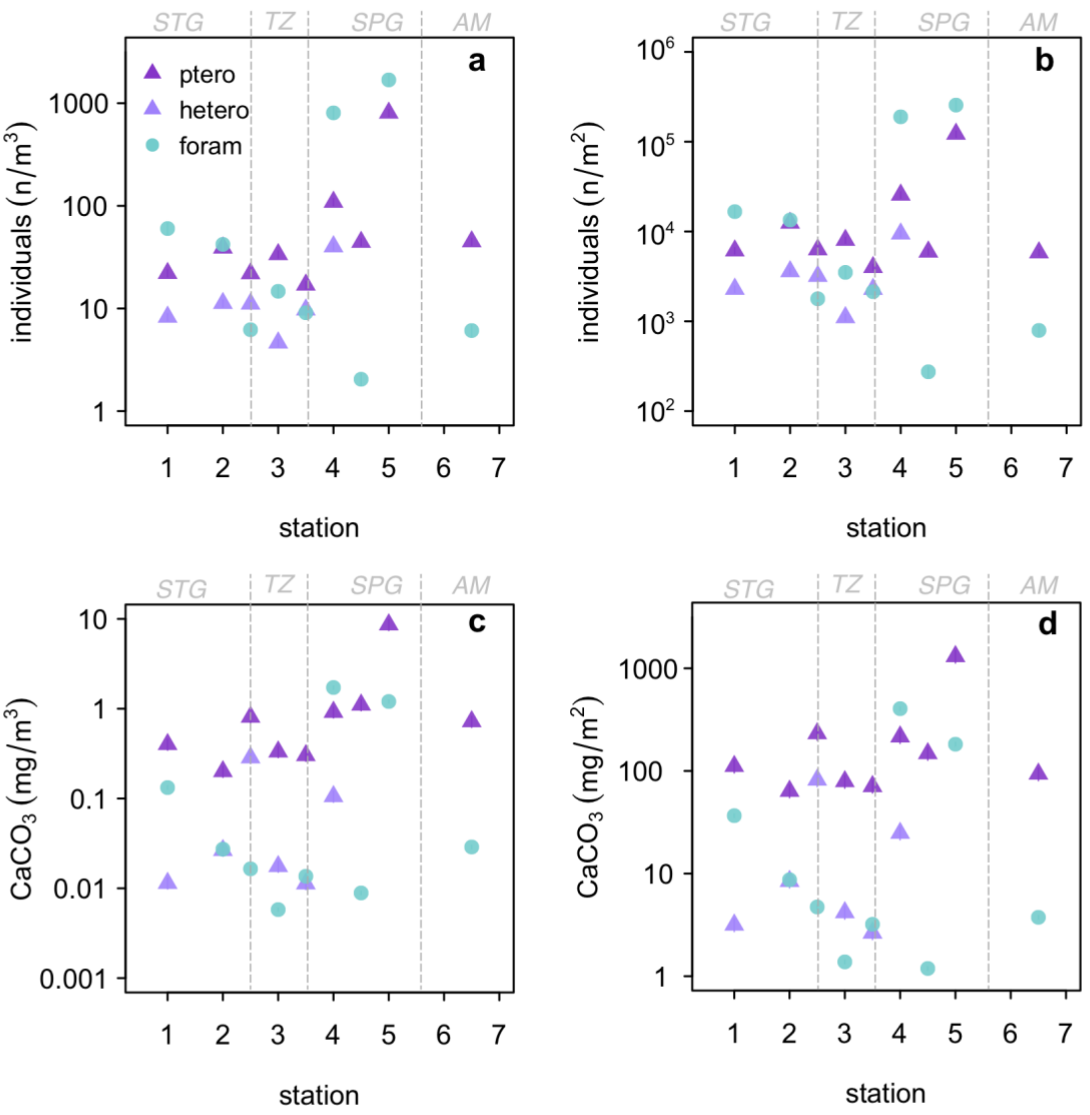

Supplementary Figure S5: Standing stock of different calcifying groups in plankton net tows across all sites **(a)** concentration of number of individuals (log scale) **(b)** vertically integrated number of individuals (log scale) and **(c)** concentration of mass of $CaCO_3$ (log scale) **(d)** vertically integrated mass of $CaCO_3$ (log scale). STG, TZ, SPG, AM are subtropical gyre, transition zone, subpolar gyre, and Alaskan margin, respectively.

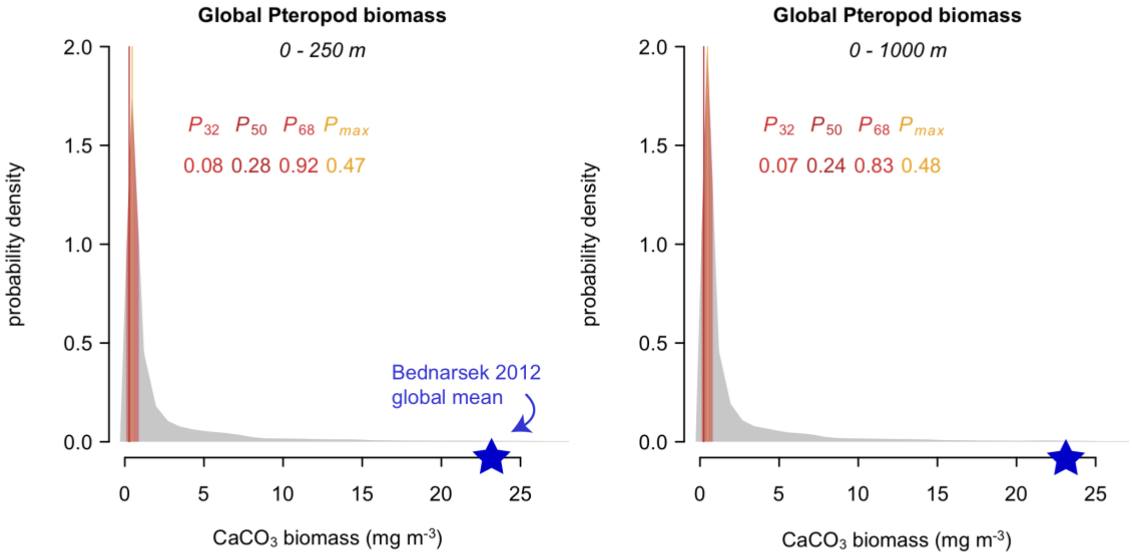

Supplementary Figure S6: Probability density of Pteropod biomass using the Global compilation of (3) showing data from the **(left)** upper 250 m, and **(right)** upper 1000 m. The 32-68% confidence interval is shaded red, the 50th percentile is shown by the red line, and the value with the highest individual probability (Pmax) is shown in orange. The 'global mean' value of 23.17 mg m-3 reported by (3) is shown by the blue star. Our analysis shows the compiled dataset is highly skewed (Skewness = 13.3), hence the mean is not a useful statistic to describe the data.

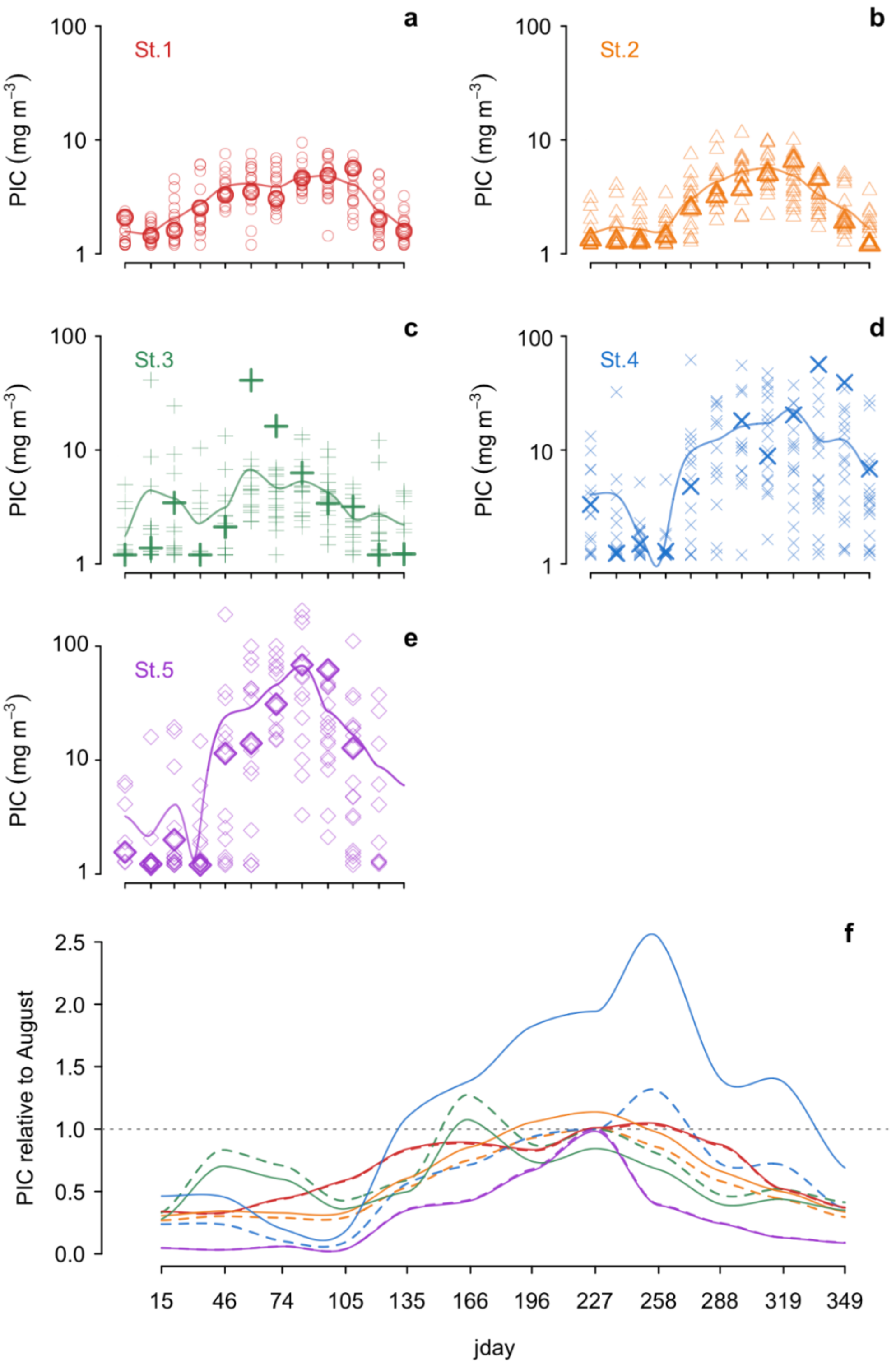

Supplementary Figure S7: (a-e) Satellite PIC (mg $CaCO_3$ m^{-3}) (4) versus julian day; small/faint symbols show monthly data for 2002-2019, large/heavy symbols show monthly data for 2017 (year of the sampling campaign of this study), and solid line shows climatology **(f)** satellite PIC versus julian day shown as a ratio relative to August; dashed line shows climatology relative to August climatology, and solid line shows climatology relative to August 2017 (year of the sampling campaign of this study).

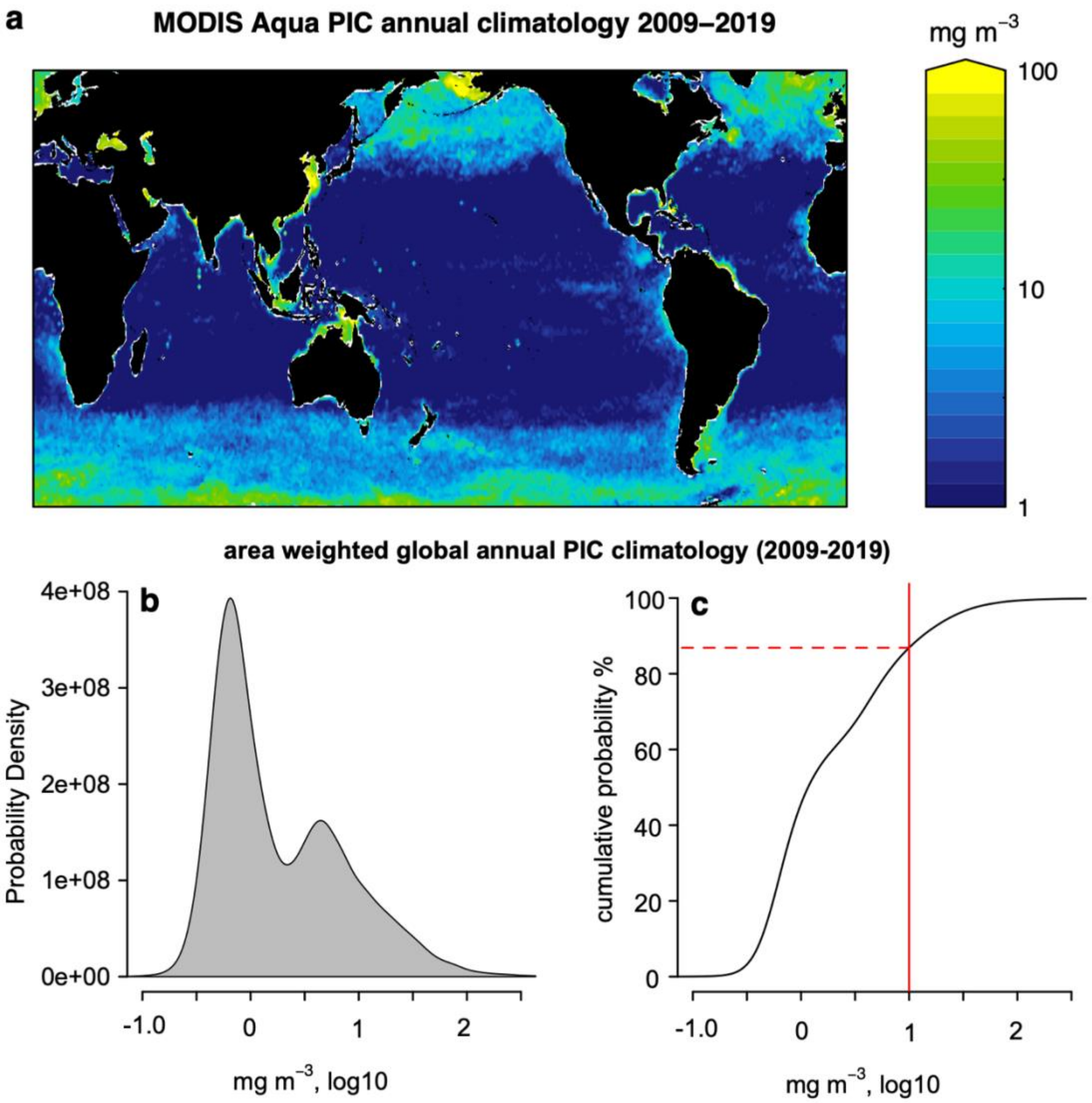

Supplementary Figure S8: **(a)** Global satellite PIC ($CaCO_3$) (4) annual climatology (2009-2020) of MODIS PIC (mg $CaCO_3$ m^{-3}; https://oceandata.sci.gsfc.nasa.gov/). Lower panels show the global (area-weighted) **(b)** probability density and **(c)** cumulative probability of this climatology.

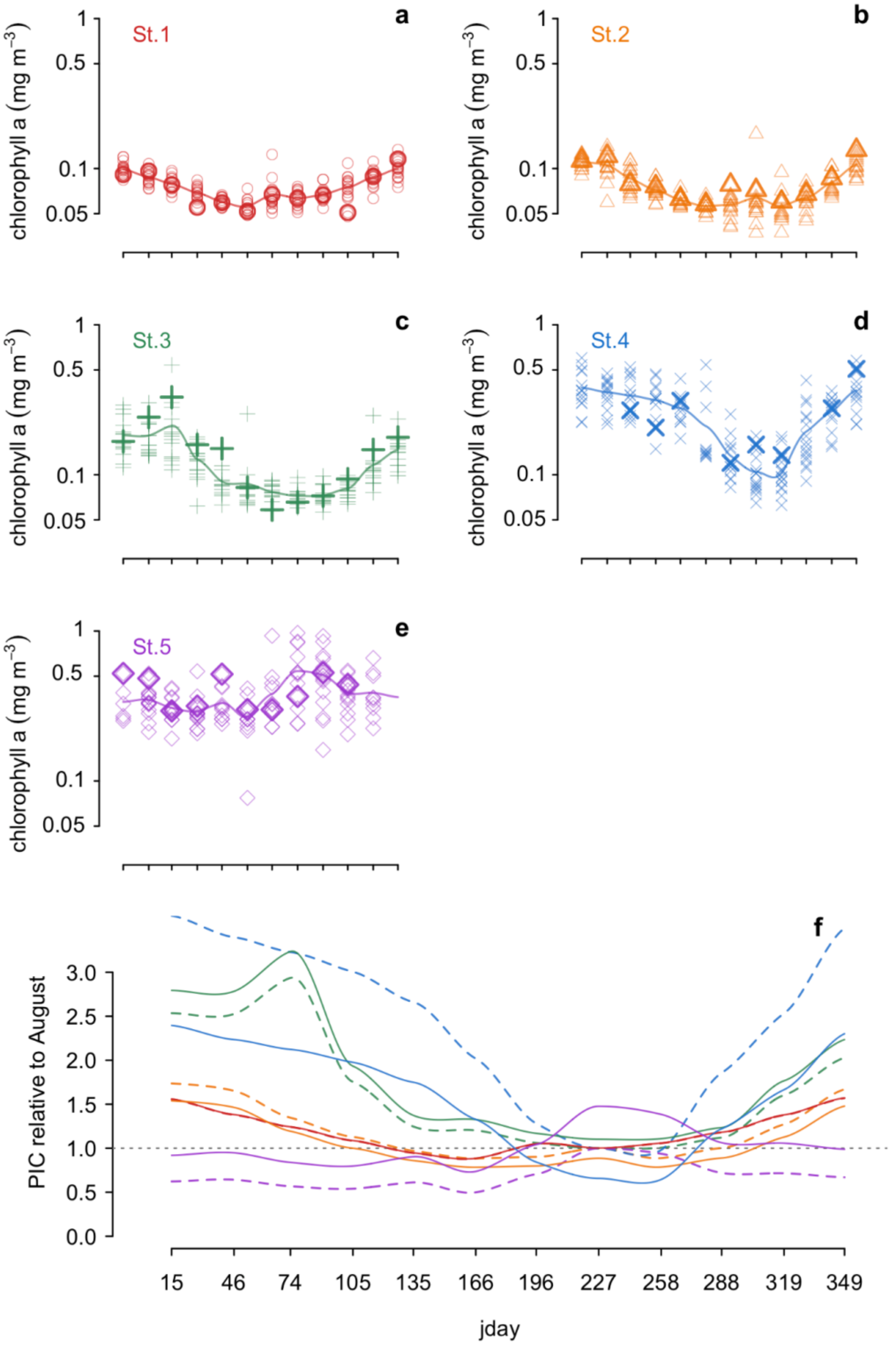

Supplementary Figure S9: (a-e) Satellite chlorophyll (mg m^{-3}) (5) versus julian day; small/faint symbols show monthly data for 2002-2019, large/heavy symbols show monthly data for 2017 (year of the sampling campaign of this study), and solid line shows climatology **(f)** satellite chlorophyll versus Julian day shown as a ratio relative to August; dashed line shows climatology relative to August climatology, and solid line shows climatology relative to August 2017 (year of the sampling campaign of this study)

Supplementary References

1. C. O'Brien *et al.*, Global marine plankton functional type biomass distributions: coccolithophores. *Earth System Science Data* **5**, 259-276 (2013).
2. E. Buitenhuis *et al.*, MAREDAT: towards a world atlas of MARine Ecosystem DATa. *Earth System Science Data* **5**, 227–239 (2013).
3. N. Bednaršek, J. Možina, M. Vogt, C. O'brien, G. Tarling, The global distribution of pteropods and their contribution to carbonate and carbon biomass in the modern ocean. *Earth System Science Data* **4**, 167-186 (2012).
4. NASA Goddard Space Flight Center, Ocean Ecology Laboratory, Ocean Biology Processing Group, Moderate-resolution Imaging Spectroradiometer (MODIS) Aqua Particulate Inorganic Carbon Data, (Greenbelt, MD, USA, 2022).
5. NASA Goddard Space Flight Center, Ocean Ecology Laboratory, Ocean Biology Processing Group. Moderate-resolution Imaging Spectroradiometer (MODIS) Aqua Chlorophyll Data, (Greenbelt, MD, USA, 2022).
